# Hydrazine‐Mediated Thermally Assisted Photocatalytic Ammonia Decomposition Over Layered Protonated Perovskites

**DOI:** 10.1002/advs.202511212

**Published:** 2025-08-29

**Authors:** Haozhe Zhang, Mengqi Duan, Shuai Guo, Renzo Leeflang, Dorottya Szalay, Jiasi Li, Jo‐chi Tseng, Simson Wu, Songhua Cai, Dharmalingam Prabhakaran, Robert A. Taylor, Yiyang Li, Shik Chi Edman Tsang

**Affiliations:** ^1^ Wolfson Catalysis Centre Department of Chemistry University of Oxford Oxford OX1 3QR UK; ^2^ Department of Applied Physics The Hong Kong Polytechnic University Kowloon Hong Kong SAR P.R. China; ^3^ Crystallography Group Diamond Light Source Diamond House Harwell Science and Innovation Campus Fermi Avenue Didcot OX11 0DE UK; ^4^ Diffraction and Scattering Division Japan Synchrotron Radiation Research Institute SPring‐8 Sayo‐gun Hyogo 679–5198 Japan; ^5^ Oxford Green Innotech Limited Oxford Business Park Oxford OX4 2HN UK; ^6^ Clarendon Laboratory Department of Physics University of Oxford Oxford OX1 3PU UK

**Keywords:** ammonia decomposition, layered perovskites, photocatalysis

## Abstract

Photocatalytic ammonia decomposition offers a sustainable route for hydrogen production, but its development is limited by low catalytic efficiency and poorly understood mechanisms. Here, a protonated layered perovskite, HPrNb_2_O_7_ (HPNO), is reported as an efficient catalyst for ammonia decomposition under mild photo‐thermal conditions. Upon exposure to NH_3_ at elevated temperatures, HPNO promotes the in situ formation and intercalation of hydrazine intermediates within its interlayer galleries, enabled by thermally generated oxygen vacancies and hydrogen bonding. Advanced characterization techniques have been applied to confirm the formation and stabilization of hydrazine. It is also shown that thermal energy prolongs charge carrier lifetimes and enhances oxygen vacancy formation, contributing to a strong photo‐thermal synergy. The stabilization of hydrazine intermediate promotes the associative mechanism, lowering the activation barrier, thus leading to an enhanced hydrogen evolution rate of 1311.2 µmol·g^−1^·h^−1^ at 200 °C under simulated solar irradiation without any noble metal co‐catalyst. This work reveals a distinct, hydrazine‐mediated reaction pathway and positions layered protonated perovskites as promising materials for efficient, solar‐driven ammonia decomposition and sustainable hydrogen generation.

## Graphical abstract

Herein, a new catalyst, protonated layered perovskite HPrNb_2_O_7_, is presented, which exhibits exceptional photocatalytic activity for solar‐driven ammonia decomposition. Its unique structure facilitates the generation of active sites while extending charge carrier lifetime with input thermal energy.

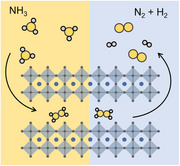

## 1 Introduction

The urgent demand for sustainable energy technologies, driven by fossil fuel depletion and environmental concerns, has intensified global interest in hydrogen (H_2_) as a clean energy carrier. Among various storage media, ammonia (NH_3_) stands out due to its high hydrogen content of 17.6 wt% and ease of liquefaction, delivering an energy density of ≈4.25 kWh L^−1^.^[^ ^1^, ^2^ ^]^ As a carbon‐free hydrogen carrier, NH_3_ offers advantages including controllable decomposition kinetics, enabling on‐demand H_2_ production for fuel cells and industrial use, and nitrogen as the sole by‐product, allowing for low‐energy purification via membrane or adsorption‐based methods.^[^ ^3^ ^]^ While thermal NH_3_ decomposition is technologically mature, it typically requires high operating temperatures (> 500 °C), leading to increased energy costs and catalyst degradation.^[^ ^4^, ^5^, ^6^ ^]^ Therefore, photocatalytic NH_3_ decomposition has emerged as a promising alternative, which can convert NH_3_ into H_2_ and N_2_ under milder conditions using solar energy. Transition metals such as Ru and Ni, widely used as active centers for thermal NH_3_ decomposition,^[^ ^7^ ^]^ have also shown promise as co‐catalysts in photocatalytic systems due to their ability to activate NH_3_ molecules at lower temperatures.^[^ ^8^ ^]^ Recently, Li et al. have reported that Ru/GaN could achieve a high hydrogen evolution rate of 3.98 mmol·cm^−2^·h^−1^ under only 5 W·cm^−2^ irradiance for NH_3_ decomposition, where the stability could reach 400 h without any degradation on activity.^[^ ^9^ ^]^ Despite these advancements, existing photocatalytic systems often rely on concentrated NH_3_ solutions and high‐intensity artificial light sources due to the poor charge carrier separation and sluggish kinetics, limiting their practical scalability and environmental compatibility.^[^ ^10^, ^11^, ^12^, ^13^ ^]^ Furthermore, the reported H_2_ evolution rates remain unsatisfactory so far, and the underlying reaction mechanisms remain poorly understood. These challenges underscore the urgent need for more efficient, solar‐responsive photocatalysts and a deeper mechanistic understanding of the NH_3_ decomposition process.

Layered perovskite oxides present an attractive platform for photocatalysis due to their structural tunability and thermal stability.^[^ ^14^, ^15^ ^]^ We have recently developed a Dion‐Jacobson type layered perovskite material, which exhibited promising photocatalytic performance for water splitting.^[^ ^16^ ^]^ The material properties can be tuned through different approaches, such as ion exchange, exfoliation, and metal doping. In the layered structure, adjacent octahedra slabs are weakly connected with each other via electrostatic force, where interlayer cations are dispersed between them, holding the whole material in an electrically neutral state. Due to the weak interlayer bonding, cations can be exchanged to tune the interlayer chemical and electronic environment, leading to unique properties and wide application of these types of materials.^[^ ^17^, ^18^, ^19^ ^]^

Here in this work, we present a thermally assisted photocatalytic NH_3_ decomposition system based on a protonated Dion‐Jacobson‐type perovskite, HPrNb_2_O_7_ (denoted as HPNO). Upon exposure to NH_3_ at elevated temperatures, HPNO facilitates the formation and intercalation of hydrazine intermediates via an associative dehydrogenation pathway. These intermediates can be stabilized within the interlayer gallery through interactions with thermally generated oxygen vacancies. A combination of advanced characterization techniques, including neutron powder diffraction (NPD), diffuse reflectance infrared Fourier transform spectroscopy (DRIFTS), X‐ray photoelectron spectroscopy (XPS), and pair distribution function (PDF) analysis, unambiguously confirms the formation and localization of N_2_H_4_. Furthermore, we show that thermal input enhances photocatalytic performance by generating oxygen vacancies and extending charge carrier lifetimes, as evidenced by time‐resolved photoluminescence (TRPL). This photo‐thermal synergy significantly boosts activity, achieving a remarkable hydrogen evolution rate of 1311.2 µmol·g^−1^·h^−1^ at 200 °C under simulated solar irradiation. These findings not only uncover a previously underexplored reaction pathway in photocatalytic NH_3_ decomposition but also establish layered protonated perovskites as a robust and versatile platform for solar‐driven hydrogen production.

## 2 Results and Discussion

### 2.1 Thermally Assisted Photocatalytic Ammonia Decomposition

**Figure 1:**
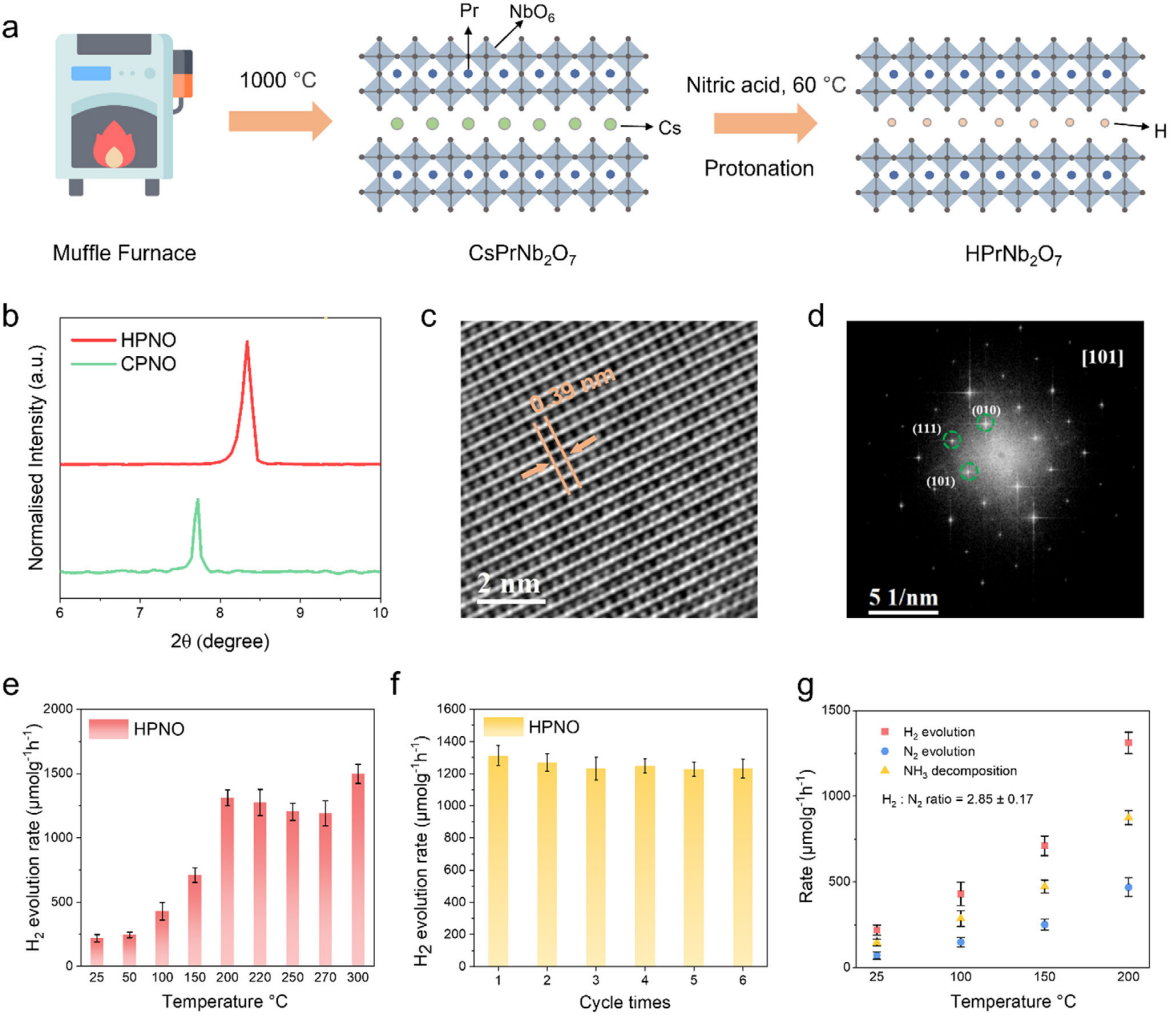
Structural characterizations and catalytic performance of HPNO. a) Schematic illustration of HPNO catalyst preparation procedure. b) XRD patterns of layered perovskite CPNO and HPNO. c) HAADF‐STEM images of HPNO. d) FFT pattern through [101] zone axis of HPNO. e) Photocatalytic ammonia decomposition activity over HPNO under different temperatures. f) The stability test of photocatalytic ammonia decomposition performance for HPNO at 200 °C for 6 cycle times (error bars represent standard deviations). g) Rate of H_2_, N_2_ evolution and NH_3_ decomposition at different temperatures.

The layered perovskite CsPrNb_2_O_7_ (denoted as CPNO) was prepared via a solid‐state synthetic method by calcining a stoichiometric mixture of Pr_6_O_11_, Nb_2_O_5,_ and Cs_2_CO_3_. Subsequent ion exchange with concentrated nitric acid at 60 °C replaced the interlayer Cs⁺ with H⁺, yielding HPNO (**Figure** **1**a). Structural evolution was monitored by powder X‐ray diffraction (XRD). Upon protonation of CPNO to form HPNO, the (001) diffraction peak shifted from 7.7° to 8.4°, reflecting a reduction in interlayer spacing due to smaller H^+^ cations (Figure 1b). High‐angle annular dark‐field scanning transmission electron microscopy (HAADF‐STEM) images of HPNO clearly show the lattice fringes with a *d* spacing of 0.39 nm for the (010) crystal plane, without any obvious dislocations or grain boundaries (Figure 1c). Corresponding Fast Fourier Transform (FFT) patterns further demonstrate the single‐crystalline nature of HPNO (Figure 1d). The light absorption properties were then evaluated using UV–vis diffuse reflectance spectroscopy, the tauc plot in Figure S1 (Supporting Information) shows that CPNO and HPNO have band gaps of 3.1 and 2.9 eV, respectively.

Photocatalytic performance for ammonia decomposition was then evaluated by measuring the hydrogen evolution rate in a closed batch reactor, illuminated with an AM1.5G solar simulator. The reaction temperature in the photocatalytic activity test was continuously monitored and regulated using a thermal controller operating in proportional‐integral‐derivative mode (Figure S2, Supporting Information). This setup maintains the bulk reaction temperature within ±0.1 °C of the setpoint by dynamically adjusting the heating input, in which the measured temperature reflects the bulk reactor environment, not the surface temperature of the catalyst particles. It should be noted that the system investigated in this work is a thermally assisted photocatalytic system rather than a photothermal catalytic system. While the terms are sometimes used interchangeably in the literature, they refer to distinct mechanisms. In our setup, the reaction temperature is controlled externally via electric heating, and not through photothermal conversion by the catalyst itself. This controlled bulk temperature promotes thermal reaction kinetics (e.g., activation of N─H bonds in NH_3_), while the light serves to generate photoexcited electrons and holes, which migrate to the catalyst surface to drive redox reactions.

Prior to testing, all catalysts were pre‐treated at 400 °C under an Ar flow to remove adsorbed NH_3_ and H_2_O species. Figure 1e illustrates that the hydrogen evolution rate increased with temperature up to ≈200 °C. Between 200 °C and 270 °C, the rate gradually declined rather than levelled off, consistent with the pressure‐induced inhibition of NH_3_ decomposition under photocatalytic conditions. Figure S3 (Supporting Information) further demonstrates that as the temperature increases, the reverse reaction of NH_3_ decomposition is more effective. Notably, at 300 °C, the activity increased again. This renewed rise is attributed to the onset of thermal (non‐photocatalytic) NH_3_ decomposition (Figure S4, Supporting Information), which becomes significant at such high temperatures and begins to compensate for the pressure‐related suppression. To further investigate the pressure effect, the internal pressure at 25, 150, 200, 225, 250, and 300 °C under an initial fill of excess NH_3_ (6 bar at 25 °C) was measured before the experiment (Figure S5a, Supporting Information), which aligns closely with the predicted values by the ideal‐gas law. Besides, we monitored the pressure over 2‐h reaction runs 25, 200, and 300 °C; however, owing to the large excess of NH_3_ and the ±1 bar resolution of the pressure gauge, no significant pressure change was observed during illumination (Figure S5b, Supporting Information). Moreover, the stability of this catalyst was assessed over six cycles (Figure 1f), with no significant loss in activity. As shown in Figure 1g, the N_2_ production rate, NH_3_ decomposition rate, and the H_2_ to N_2_ ratio at different temperatures are all provided, in which the ratio H_2_ to N_2_ ratio is estimated around 2.9, close to the theoretical value of 3. As shown in Figure S6 (Supporting Information), the quantum efficiency (QE) has been measured for HPNO under monochromatic illumination. It demonstrates how irradiation wavelengths influence NH_3_ conversion, giving the highest QE at 385 nm (Note S1, Supporting Information). In addition, XRD patterns (Figure S7, Supporting Information) remained unchanged after the stability test, confirming the robustness of the HPNO catalyst. Control experiments under Ar showed no H_2_ production under light, verifying that the activity originates solely from NH_3_ decomposition. Also, no H_2_ production could be observed from 6 bar of NH_3_ at 200 °C without irradiation, further confirming the photocatalytic nature of this system. A deuterated analogue (DPrNb_2_O_7_) was synthesised via repeated D_2_O exchange of HPrNb_2_O_7_. Then, the NH_3_ decomposition reaction was conducted on this material under AM 1.5G illumination at 200 °C. Mass spectrometry of the evolved gases detected only H_2_, with no D_2_ or HD (Figure S8, Supporting Information), indicating that the interlayer protons do not contribute to hydrogen evolution, and NH_3_ is the only hydrogen source under our reaction conditions. To gain deeper insight into the thermal‐assisted photocatalytic system, thermal‐only NH_3_ decomposition was investigated in the absence of light (Figure S9, Supporting Information). All catalysts displayed enhanced activity with increasing temperature due to more favorable reaction kinetics. Notably, HPNO initiated NH_3_ decomposition at 400 °C, and achieved an NH_3_ conversion of 43.4% at 550 °C even under a high weight hourly space velocity (WHSV) of 30000 mL g_cat_^−1^ h^−1^. In contrast, CsPrNb_2_O_7_ only showed a conversion of 12.5% at 550 °C, highlighting the critical role of the perovskite protonation. Furthermore, a light‐concentrated furnace was used to mimic a solar furnace (Figure S10, Supporting Information), showing that stable and efficient ammonia decomposition activity at 200 °C could be maintained solely by the concentrated light without any other external heating devices.

**Figure 2:**
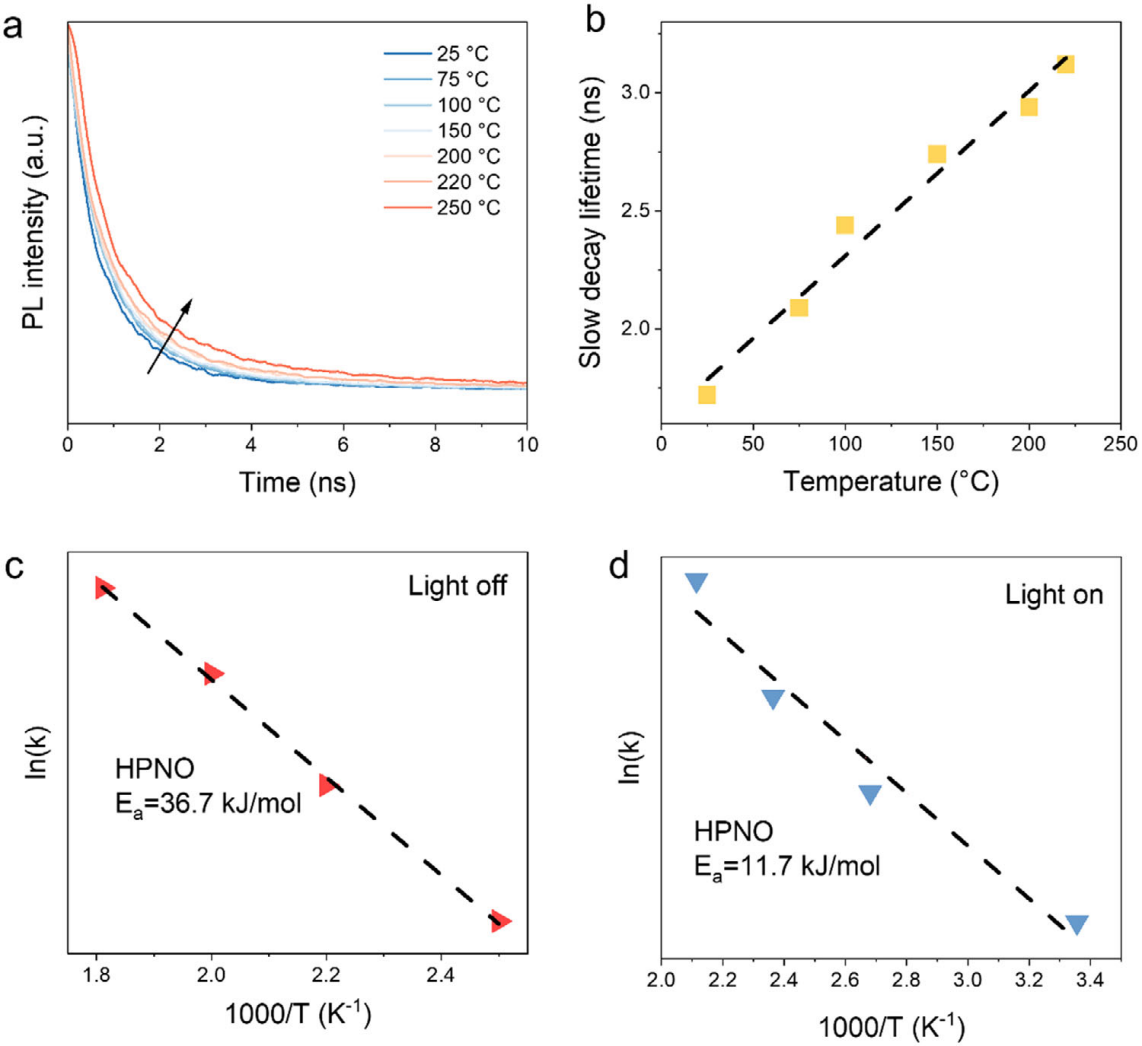
Kinetic study of photocatalytic ammonia decomposition. a) TRPL spectra of HPNO under temperatures from 25 °C to 250 °C, where the excitation wavelength is 430 nm. b) Charge carrier lifetime versus temperature. c) Arrhenius plots of HPNO for thermal ammonia decomposition. d) Arrhenius plots of HPNO for photocatalytic catalysis conditions.

It is widely accepted that the separation of the photogenerated charge carriers plays a vital role in photocatalytic systems. Therefore, TRPL spectroscopy was then used to probe the photogenerated charge carrier dynamics at various temperatures (**Figure** **2**a; Table S1, Supporting Information). Two decay components could be identified by fitting the TRPL spectra in Figure S11 (Supporting Information): a fast bulk recombination and a slower component associated with surface or interlayer recombination processes. As shown in Figure 2b, the average lifetime increased linearly with temperature, indicating suppressed recombination, presumably due to the exciton delocalization facilitated by thermal energy.^[^ ^20^, ^21^, ^22^, ^23^ ^]^ This enhancement also contributes to the observed photo‐thermal synergy in this NH_3_ decomposition system. In the protonated layered perovskite system, photogenerated electrons and holes are confined within the 2D NbO_6_ octahedral slabs. Rapid recombination rate of charge carriers would shorten their lifetimes, avoiding them from migrating to surface active sites, such as surface oxygen vacancies, to drive the ammonia decomposition reaction. In contrast, a longer carrier lifetime suggests suppressed recombination, allowing both electrons and holes sufficient time to reach the surface and participate in the following redox processes. In perovskite photocatalysts, efficient separation and migration of the photogenerated charge carriers are therefore critical for high photocatalytic activity. Figure S12 (Supporting Information) demonstrates a positive correlation between the increased TRPL lifetime and enhanced photocatalytic performance, supporting the role of charge dynamics in the overall reaction efficiency.

To probe the activation barrier associated with these catalysts, Arrhenius analysis was first performed for thermal catalytic conditions (Figure 2c), where the linear fitting of reaction rate versus 1/T. The apparent activation energy (*E*_a_) estimated from this plot corresponds to the *E*_a_ in the rate‐determining step in the overall chemical process. The apparent *E*_a_ for HPNO was determined to be 36.7 kJ·mol^−1^. Under photocatalytic conditions, the *E*_a_ values dropped significantly to 11.7 kJ·mol^−1^ for HPNO (Figure 2d). Such a substantial reduction in *E*_a_ indicates a distinct reaction mechanism under photocatalytic conditions, compared to purely thermal pathways, inspiring further mechanistic investigations into the photocatalytic NH_3_ decomposition process over HPNO perovskites.

### 2.2 Mechanistic Investigations

To further probe the origin of the observed photo‐thermal enhancement and the role of intermediates, mechanistic studies were performed. Early studies have proposed photocatalytic NH_3_ decomposition pathways over Pt/TiO_2_, where the photogenerated holes oxidise adsorbed NH_3_ molecules into amide radicals and protons. These subsequently form hydrazine (N_2_H_4_), which then decomposes into N_2_ and H_2_.^[^ ^11^ ^]^ Density functional theory (DFT) supports this associative pathway via H_2_N‐NH_2_, which has a much lower activation energy (≈59.2 kcal·mol^−1^) compared to the dissociative route (≈235.7 kcal·mol^−1^).^[^ ^24^ ^]^ However, direct experimental evidence for this intermediate remains limited. In layered perovskite systems, such as HPNO, it is anticipated that reaction intermediates may access and be stabilized within the interlayer galleries via hydrogen bonding or interactions with defects. To investigate this possibility, a wide range of in situ characterization techniques was employed to monitor the formation, stabilization, and transformation of intermediates.

**Figure 3:**
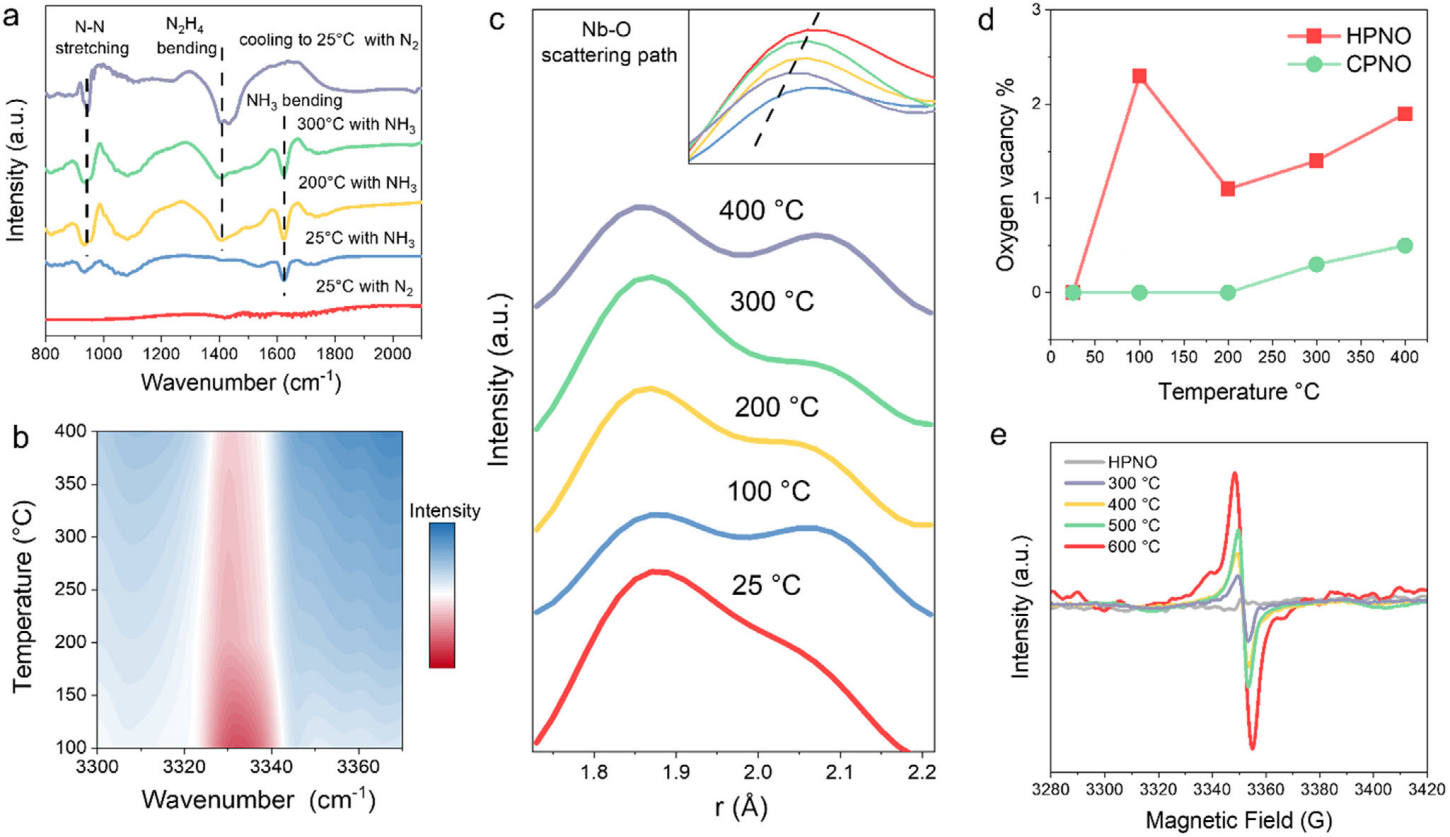
Ammonia decomposition process within HPNO. a) DRIFTS study of the hydrazine formation process, where HPNO is heated under NH_3_ flow. b) DRIFTS study of NH_3_ decomposition under different temperatures over HPNO. (c) XPDF of Nb‐O bond heated from 25 to 400 °C under ammonia gas. d) Oxygen vacancy variation as temperature increases under NH_3_, which is determined by the refinement of XPDF. e) EPR pattern of HPNO after heating at different temperatures with NH_3_ flow.

DRIFTS was used to track NH_3_ conversion on HPNO catalyst. As shown in **Figure** **3**a, no characteristic vibrational signals were observed in the 800–2100 cm^−1^ range under N_2_ at 25 °C. Upon introduction of NH_3_, a band at 1623 cm^−1^ emerged, attributed to the bending mode of NH_3_.^[^ ^25^ ^]^ Notably, with increasing temperature up to 300 °C, new bands appeared progressively at 1427 and 948 cm^−1^, which align with the *δ*(H‐N‐H) bending and *ν*(N‐N) stretching modes of N_2_H_4_, respectively.^[^ ^20^ ^]^ After cooling and N_2_ purging, the NH_3_ band vanished while the N_2_H_4_ signals persisted, indicating stabilization of N_2_H_4_ species on HPNO. A DRIFTS heat map (Figure 3b) revealed temperature‐dependent consumption of NH_3_ via a progressively diminishing N‐H stretching band at 3320–3340 cm^−1^, further supporting NH_3_ conversion. The remaining signal can be assigned to N‐H stretching of N_2_H_4_, which decreases upon further temperature increase due to its decomposition.

In light of the DRIFTS results, X‐ray pair distribution function (XPDF) analysis was then used to probe the dynamic structural changes of the layered HPNO perovskite associated with NH_3_ conversion at various temperatures. As shown in Figure 3c, the characteristic Nb‐O peak at 1.9 Å broadened and diminished at 100 °C due to lattice vibration and oxygen vacancy formation.^[^ ^26^, ^27^, ^28^ ^]^ Notably, a partial recovery at 200°C was observed, linked to NH_3_ coordination with undercoordinated Nb sites via Lewis acid‐base interactions.^[^ ^29^ ^]^ Through the XPDF refinements (Figure S13, Supporting Information), we found that such interactions can refill the oxygen vacancies, thereby restoring Nb‐O symmetry from *NH_3_ compensation (Figure 3d).^[^ ^30^ ^]^ Above 300 °C, intensity again declined (Figure 3c), likely from desorption of N‐N coupled products (e.g., N_2_H_4_ or N_2_). These structural changes provide real‐time evidence of reaction intermediate evolution within the layered HPNO perovskite. XPDF refinements of both HPNO and CPNO were also performed to quantify the oxygen vacancy evolution within their layered framework (Figure S13, Supporting Information). While HPNO showed progressive vacancy formation and dynamic structural responses, CPNO remained structurally inert, with minimal vacancy formation up to 400 °C (Figure 3d). This suggests that Cs^+^ interlayer cations stabilise the layered lattice, hindering NH_3_ adsorption and conversion. Moreover, the simple Rietveld refinement of in situ Synchrotron XRD of CPNO (Figure S14, Supporting Information) shows that even under air conditions, increasing temperature could still facilitate oxygen vacancy formation, and there is no vacancy refilling under the air. These findings are reinforced by temperature‐programming surface reaction (TPSR) experiments (Figure S15, Supporting Information), where HPNO displayed a marked increase in NH_3_ consumption at 300 °C due to thermally induced vacancies. In contrast, CPNO exhibited suppressed uptake, indicating limited NH_3_ access in the absence of vacancy generation.

TPSR analysis at 200 °C (Figure S16, Supporting Information) revealed that the instantaneous NH_3_ consumption rate over HPNO increased gradually from 0 to 1700 s. This steady rise corresponds to an induction period, during which oxygen vacancies are progressively formed, increasing the number of active sites available for NH_3_ decomposition. The rate eventually plateaus, indicating that vacancy formation is the rate‐limiting step, and a dynamic equilibrium is established between vacancy generation, NH_3_ adsorption, and subsequent decomposition. To directly confirm oxygen vacancy formation, electron paramagnetic resonance (EPR) spectroscopy was performed. HPNO was pre‐treated in an NH_3_ flow at various temperatures before EPR measurements. A characteristic signal at g ≈ 2.005 was observed in NH_3_‐treated HPNO (Figure 3e), consistent with vacancies adjacent to Nb^5+^ sites.^[^ ^31^ ^]^ The signal increased gradually with temperature up to 500 °C, confirming thermally activated defect generation, which agrees with the results shown above. Notably, a sharp rise at 600 °C accompanied by hyperfine splitting suggests the formation of metastable vacancy clusters, likely stabilized by substitutional N^3−^ doping from NH_3_ decomposition.

Having established that N_2_H_4_ forms and is stabilized on HPNO at elevated temperatures, further efforts have been made to elucidate the position and local structure of the N_2_H_4_ species. To study the static N_2_H_4_ intermediate, the pristine HPNO sample was treated in an NH_3_ flow at 300 °C in order to trap the N_2_H_4_ species. As indicated by the DRIFTS results mentioned earlier, N_2_H_4_ species formed at this temperature can be maintained upon cooling and NH_3_ removal. For comparison, HPNO was also treated in an NH_3_ flow at 600 and 800 °C, respectively. These treated samples are denoted as HPNO‐300, HPNO‐600, and HPNO‐800, respectively.

**Figure 4:**
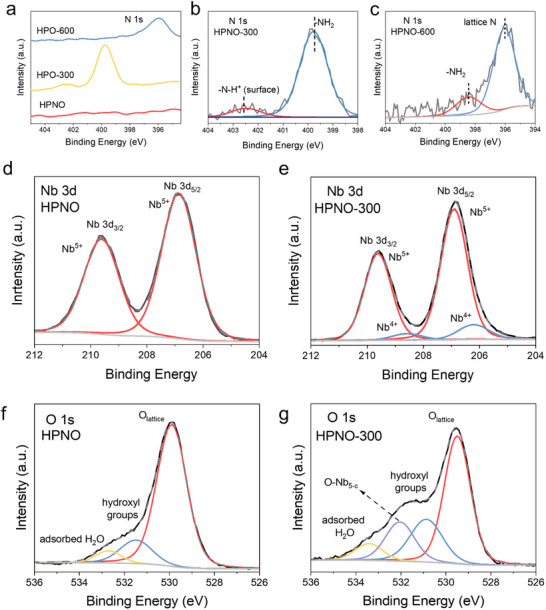
XPS analysis on HPNO layered perovskite. a) N 1s XPS spectra of HPNO, HPNO‐300, and HPNO‐600. High resolution N 1s XPS spectra of b) HPNO‐300 and c) HPNO‐600. Grey line: raw data; Blue and Red lines: fitted data. Nb 3d XPS spectra of d) HPNO and e) HPNO‐300. O 1s XPS spectra of f) HPNO and g) HPNO‐300.

XRD analysis showed a shift in the (001) reflection from 8.4° (pristine HPNO) to 8.1° (HPNO‐300), indicating slight interlayer expansion presumably due to N_2_H_4_ intercalation. HPNO‐600 shows a very similar XRD pattern to that of HPNO‐300. However, treatment at 800 °C led to structural collapse and complete loss of the characteristic (001) peak, confirming decomposition of the layered framework (Figure S17, Supporting Information).^[^ ^32^ ^]^ XPS was also used to study the chemical environment of these NH_3_‐treated materials (**Figure** **4**a). For HPNO‐300, a prominent peak at 399.7 eV appeared, assigned to N_2_H_4_ species (Figure 4b).^[^ ^33^, ^34^, ^35^, ^36^ ^]^ In contrast, HPNO‐600 showed the disappearance of the hydrazine peak and emergence of signals at 396.1 and 398.9 eV, consistent with substitutional N^3−^ doping and residual N_2_H_4_ (Figure 4c). This further confirms that N_2_H_4_ can be stabilized up to 300 °C, but decomposes at higher temperatures, leading to substitutional nitrogen doping into the perovskite lattice, agreeing with the results demonstrated above. Moreover, the Nb 3*d* spectrum of HPNO shows Nb 3*d*_3/2_ and Nb 3*d*_5/2_ peaks located at 209.8 and 207.1 eV, indicating its valence feature of Nb^5+^ (Figure 4d). In HPNO‐300, there are two extra Nb 3d peaks that appear at 208.0 and 204.9 eV attributed to Nb^4+^ (Figure 4e). Deconvolution of the O 1*s* spectrum for HPNO exhibits three O species, which can be assigned to the lattice O (O_lattice_), surface hydroxyl groups (O‐H), and adsorbed H_2_O, respectively (Figure 4f). While an additional component can be observed for HPNO‐300 at 531.8 eV, indicating oxygen defect formation (Figure 4g),^[^ ^31^, ^37^, ^38^ ^]^ which is consistent with the XPDF and EPR analyses. While oxygen vacancies themselves do not contribute photoelectrons and thus cannot be directly observed in XPS, their formation leads to structural and electronic changes in the surrounding lattice. Specifically, the formation of an oxygen vacancy results in adjacent Nb atoms becoming five‐coordinated, rather than the typical six‐coordinated Nb in NbO_6_ octahedra. This local distortion then influences the binding energy of nearby oxygen species, which is labelled as O‐Nb_5c_. Thermogravimetric analysis (TGA) was employed to gain more information of HPNO‐300 and HPNO‐600. HPNO‐300 shows a significant mass loss (≈2.5 wt%) initiating at 400 °C, which can be attributed to thermal decomposition of N_2_H_4_ (Figure S18, Supporting Information). In contrast, HPNO‐600 demonstrates an increase in mass (≈1.5 wt%) above 300 °C, consistent with the oxidative substitution of lattice‐incorporated N^3−^ anions by O^2−^. Both samples underwent further mass loss above 550 °C due to oxygen vacancy formation. These results corroborate that HPNO‐300 contains intercalated N_2_H_4_, while HPNO‐600 features substitutional N doping. UV–vis spectroscopy provided more direct evidence for the presence of N_2_H_4_ in HPNO‐300. Upon condensation with *para*‐(dimethylamino)benzaldehyde, a colored azine complex forms with an absorption band at 400–450 nm.^[^ ^39^ ^]^ Increasing the mass of HPNO‐300 led to proportional absorbance enhancement (Figure S19, Supporting Information), confirming the presence of N_2_H_4_ in HPNO‐300.

**Figure 5:**
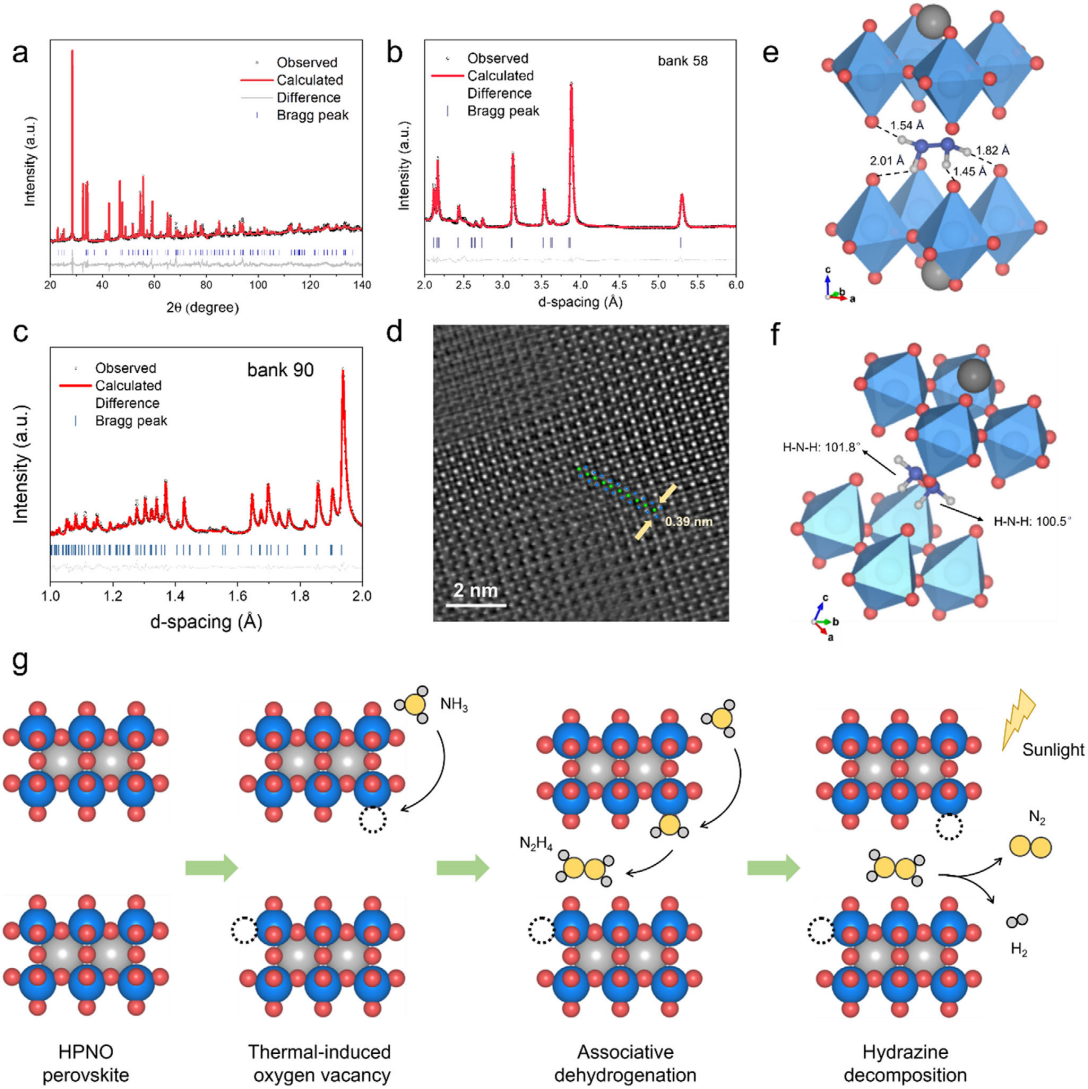
Structural characterizations of HPNO‐300. a) XRD Rietveld refinement of HPNO at 298 K, R_wp_ = 9.6%. NPD joint Rietveld refinement of HPNO at 298 K, R_wp_ = 1.9%, at b) bank 58 and c) bank 90. d) HAADF‐STEM images of HPNO‐300 with FFT pattern through [001] zone axis. e,f) The refined structure of HPNO‐300 (the proton between the interlayer oxygen was hidden in order to give a more comprehensive view of N_2_H_4_). The dashed line represents the NH···O interaction. g) Schematic illustration of the thermally assisted photocatalytic NH_3_ decomposition mechanism over the HPNO catalyst.

To further elucidate the structure of intercalated HPNO‐300, Rietveld refinement of XRD data was performed (**Figure** **5**a). The material crystallizes in a *P*1 space group with lattice parameters *a* = 3.85151 Å, *b* = 3.86932 Å, *c* = 10.57143 Å, and *α* = *β* = *γ* = 90°, comprising corner‐sharing NbO_6_ octahedra arranged in double‐layer slabs stacked along the *c*‐axis. To precisely determine the location of light elements such as N and H, NPD was employed due to its high sensitivity to small atomic displacements and strong contrast for oxygen and nitrogen.^[^ ^40^ ^]^ The NPD refinement of the *P*1 model (Figure 5b,c) confirmed that N_2_H_4_ molecules occupy the center of the interlayer galleries, with a N‐N distance of 0.14 nm, consistent with that in molecular N_2_H_4_. Hydrogen bonding interactions between the N‐H groups of N_2_H_4_ and lattice oxygen atoms (Figure 5e,f) slightly lengthen the N‐H bond to 0.11 nm and compress the H─N─H bond angles to 101.8° and 100.5°, compared to free N_2_H_4_. This distortion arises from the restricted interlayer spacing and the altered lone pair‐bond pair repulsion balance.

Moreover, HAADF‐STEM images in Figure 5d; Figures S20 and S21 (Supporting Information) further confirmed the high crystallinity of HPNO‐300, as indicated by sharp FFT patterns and well‐defined lattice fringes. Additionally, SEM‐EDS mapping (Figure S22, Supporting Information) verified the homogeneous distribution of Pr, Nb, O, and N elements throughout the material. Complementary insights were obtained via ^14^N solid‐state nuclear magnetic resonance (ssNMR) spectroscopy. The NMR spectrum (Figure S23, Supporting Information) reveals a substantial pattern of spinning sidebands. These sidebands are caused by the presence of anisotropic interactions, whose source can be quadrupolar coupling effects but as well as the spin‐spin interactions with protons.^[^ ^41^, ^42^ ^]^ The breakdown of the ideal symmetry of the surroundings is due to the different NH···O interaction distance between N_2_H_4_ hydrogen and perovskite lattice oxygen (Figure 5e).^[^ ^43^, ^44^ ^]^ These findings support the conclusion that N_2_H_4_ occupies well‐defined, yet distorted, interstitial positions within the layered structure, which is consistent with the XRD and NPD results. Taken together, these findings confirm that NH_3_ can be selectively converted to N_2_H_4_ over HPNO at elevated temperatures, with the resulting N_2_H_4_ stably intercalated between the layers. This stabilization is facilitated by hydrogen bonding, promoting the associative NH_3_ decomposition pathway. This mechanism underpins the enhanced activity observed in thermal‐assisted photocatalytic NH_3_ decomposition over layered HPNO perovskites. To investigate the role of light in hydrazine activation, we exposed the N_2_H_4_‐intercalated HPNO‐300 sample to intense illumination using a 300 W Xe lamp, followed by synchrotron X‐ray diffraction and Rietveld refinement (Figure S24, Supporting Information). The resulting diffraction patterns showed no detectable N_2_H_4_ signature in the interlayer region, while a clear increase in oxygen vacancy concentration was observed. These results suggest that N_2_H_4_ undergoes photocatalytic decomposition under strong light irradiation. To quantify the light dependence of this photocatalytic system, we measured H_2_ evolution rates under varying irradiances (Figure S25, Supporting Information), from dark conditions up to 100 mW cm^−2^. A clear, irradiance‐dependent increase in H_2_ evolution rate was observed, confirming that photon flux plays a key role in driving the overall NH_3_ decomposition process.

Figure 5g illustrates the proposed photo‐thermal NH_3_ decomposition mechanism derived from these findings. Under thermal activation, oxygen vacancies are generated within HPNO. These vacancies act as active sites for the NH_3_ decomposition process, where incoming NH_3_ molecules adsorb onto these points to replenish these vacancy sites, as confirmed by XPDF analysis (Figure 3c). The adsorbed *NH_3_ intermediates subsequently undergo associative dehydrogenation, forming N_2_H_4_ as a reaction intermediate. NPD refinements (Figure 5b,c) reveal that the HPNO perovskite lattice effectively stabilizes N_2_H_4_ within its layered structure. Under simulated solar irradiation, photoexcitation generates electron‐hole pairs, which migrate to the catalyst surface to drive N_2_H_4_ decomposition. Concurrently, thermal input extends the lifetimes of photogenerated carriers, as demonstrated by TRPL measurements (Figure 2a), synergistically boosting the overall ammonia decomposition activity.

## 3 Conclusion

In this study, we have developed a protonated layered perovskite, HPrNb_2_O_7_, as an efficient and noble‐metal‐free catalyst for photocatalytic ammonia decomposition under mild conditions. We demonstrate that upon exposure to NH_3_ at elevated temperatures, hydrazine is generated via an associative dehydrogenation mechanism and stably intercalated within the interlayer gallery of HPNO. This intermediate is stabilized through hydrogen bonding and interactions with thermally generated oxygen vacancies. Comprehensive characterization techniques have been used to confirm the presence and structural environment of the intercalated hydrazine species. Our results also show that moderate thermal input extends lifetimes of the photogenerated charge carriers and promotes vacancy formation, leading to a strong photo‐thermal synergy. As a result, a superior H_2_ evolution rate of 1311.2 µmol·g^−1^·h^−1^ is demonstrated in this system at 200 °C (Table S2, Supporting Information). This work reveals a mechanistically distinct pathway for NH_3_ decomposition over layered perovskites and unravels the local structure and interactions of the intercalated hydrazine intermediate. These insights not only deepen our understanding of NH_3_ activation but also highlight the potential of other layered perovskites as robust and scalable platforms for sustainable hydrogen evolution under solar‐driven conditions.

## Conflict of Interest

The authors declare no conflict of interest.

## Author Contributions

H.Z. prepared and tested the catalysts and analyzed the results. M.D. performed a SEM study. F.O. and P.M. performed neutron diffraction experiments. S.G. and S.C. carried out microscopic studies. R.L. and H.Z. performed and analyzed DRIFTS experiments. D.S. performed ICP‐MS measurements. J.L. analyzed SXRD results. J.‐c.T. carried out XPDF experiments. H.Z., Y.L., and D.P. performed the light furnace experiments. Y.L. and R.A.T. performed and analyzed the TRPL measurements. H.Z., Y.L., and S.W. wrote the paper in discussion with all authors. S.C.E.T. supervised the overall project.

## Supporting information



Supporting Information

## Data Availability

The data that support the findings of this study are available from the corresponding author upon reasonable request.
