# Supplementary material for: Hydrazine‐Mediated Thermally Assisted Photocatalytic Ammonia Decomposition Over Layered Protonated Perovskites
Source: Adv Sci (Weinh). 2025 Aug 29;12(42):e11212. doi: 10.1002/advs.202511212 (PMC12622521; doi:10.1002/advs.202511212)
Supplement: Supplementary file 1 — Supporting Information [file ADVS-12-e11212-s001.docx]

**Supplementary Information for**

**Hydrazine-Mediated Thermally Assisted Photocatalytic Ammonia Decomposition over Layered Protonated Perovskites**

H. Zhang et al.

**This file includes:**

Methods

Supplementary Figures (S1-S25) and Tables (S1-S2)

Note S1

References

**Methods**

**Preparation of layered perovskite CsPrNb_2_O_7_ (CPNO)**

It was synthesised by both solid-phase and aqueous-phase methods, which are referenced to the previous study. Pr_6_O_11_ (Sigma-Aldrich, >99.996%) and Nb_2_O_5_ (Sigma-Aldrich, >99.9%) was dried at 900 °C before being used. After that, suitable quantity of metal oxides was ground together with Cs_2_CO_3_ (Sigma-Aldrich, >99%). A 50% excess amount of Cs_2_CO_3_ was added to compensate for the loss during calcination because of its volatility at high temperature. The mixtures were then heated at 600 °C for 12 h and re-ground. Samples of resulting powder was heated with air flow for 12 h at 1000 °C. Finally, all the collected layered perovskite samples were washed by ultrasonicating with deionised water for several times to remove any remaining Cs oxides and then dried for 24 h at 60 °C in a vacuum oven

**Preparation of HPrNb_2_O_7_ (HPNO)**

The crystalline layered perovskites CsPrNb_2_O_7_ were stirred with 6 M of HNO_3_ solution at 60 °C for 7 days. Cs^+^ in the interlayer space was replaced by protons. The collected powder samples were washed with deionised water until the pH = ~7. Then the resulting product HPrNb_2_O_7_ was dried for 48 h at 60 °C in a vacuum oven.

**Preparation of HPNO-300**

Then 100mg of HPrNb_2_O_7_ was heated at 300 °C for 6 h under NH_3_ flow (flow rate = 10mL/min). The collect sample was washed with deionised water, then dried for 48 h in a vacuum desiccator at 60 °C.

**X-ray diffraction (XRD)**

XRD patterns were performed on a PANalytical X’Pert Pro diffractometer using Cu anode operating at 40 kV and 30 mA with a step size of 0.004°. Perovskite samples were dusted onto a glass. All structures were refined by Rietveld analysis using j-Edit and TOPAS program.

***In-situ* synchrotron powder X-ray diffraction (*in-situ* SXRD)**

*In-situ* SXRD data were collected on Beamline TPS 19A, National Synchrotron Radiation Research Centre, Taiwan, China. The energy of the incident X-ray flux was set at 28.5 keV with a calibrated wavelength 0.43503 Å. High-resolution SXRD data of all samples were achieved by using the MYTHEN 18K detector in the 2*θ* range 0 – 90° with 0.001° data binning. The capillary was mounted to a sample holder and heated up at different temperature from 273 K to 427 K with a ramping rate of 5 K/min.

**X-ray photoelectron spectroscopy (XPS)**

Samples were prepared by dispersing the powders in ethanol and depositing them onto a silicon wafer which were then heated to remove the solvent. XPS measurements were performed at 20 °C K under vacuum. All spectra were obtained using a fixed photon energy of 1600 eV. The binding energy was then calibrated with respect to the C 1s peak (binding energy = 284.8 eV) as an internal standard.

**Ultraviolet-visible diffuse reflectance spectroscopy (UV-vis)**

UV-vis absorption spectra were obtained by a reflection method using a PerkinElmer Lambda UV-visible spectrometer at room temperature. 50 mg of each sample was loaded and pressed onto a sample holder and UV-vis spectra were recorded within the wavelength range of 300 - 800 nm.

The optical bandgap energies of the materials were determined through Tauc plot analysis derived from UV-vis spectroscopy measurements. This methodology involves two critical analytical steps: First, the measured reflectance spectra were converted into absorption profiles using the Kubelka-Munk transformation:

$$F(R_{\infty})=\frac{K}{S}=\frac{(1-R_{\infty})^{2}}{2R_{\infty}}$$

where *R*∞​ represents the relative diffuse reflectance of a semi-infinite sample layer, while *K* and *S* correspond to the absorption and scattering coefficients, respectively. Subsequently, the Tauc relationship was applied to identify the nature of electronic transitions:

$$(F(R_{\infty})\cdot h\nu)^{\frac{1}{\gamma}}=B(h\nu-E_{g})$$

Here, *hν* denotes photon energy, *E*g​ is the bandgap energy, *B* is a material-specific proportionality constant, and *γ* characterizes the transition type (*γ*=2 for direct bandgap semiconductors, *γ*=1/2 for indirect transitions). The characteristic bandgap energy was determined by extrapolating the linear region of the Tauc plot – the photon energy axis intercept of the best-fit line to the steeply rising absorption edge provides the *E*g​ value.

**Thermogravimetric analysis (TGA)**

TGA was performed on a TA Instruments TGA Q600 under an airflow of 100 ml/min. A sample with an exact amount (10 - 25 mg) was added into an alumina pan and heated from room temperature to 900 °C using the heating rate of 10 °C /min. A corresponding TGA curve together with its first derivative was collected simultaneously.

**Temperature-programming surface reaction (****TPSR)**

TPSR was performed on a Quantachrome ChemBET Pulsar analyser equipped with a cold trap. The detection of desorbed molecules was done by the thermal conductivity detector, and the current was set to 150 mA. Approximately 100 mg of catalyst was loaded into a double-walled quartz reactor equipped with Swagelok™ gas fittings at both ends to ensure leak-tight reactant flow. The reactor design enabled controlled gas-phase delivery through distinct flow paths: reactant gases entered the inner annulus (flowing downward from top to bottom), permeated through the catalyst bed, and exited via the outer annulus (flowing upward from bottom to top). Effluent gases were analysed in real-time using an in-line thermal conductivity detector (TCD). Temperature regulation was achieved via a programmable tube furnace, with synchronized recording of temperature profiles and TCD signals to correlate thermal conditions with catalytic activity.

The sample was first pre-treated for 1 h under pure He gas to remove any impurity or water from the surface. The temperature was maintained at 25 °C until a stable TCD baseline was obtained. After switching the gas to NH_3_, analysis was conducted while the temperature was increased to 700 °C at a ramp rate of 45 °C/min.

**Diffuse reflectance infrared Fourier transform spectroscopy (DRIFTS)**

All DRIFTS data was collected with a multiple-reflection ATR accessory (custom-modified GladiATR from PIKE Technologies) in a Varian 680-IR spectrometer controlled by Resolutions Pro software. Fresh samples were loaded onto the sample holder, and the sample was flushed with Ar for 30 min to clean the surface under required temperature. After collecting background spectra, NH_3_ gas was passed through the sample holder at 10 mL min^−1^ and sample spectra were then recorded. Each spectrum was scanned 64 times.

**^14^N solid-state nuclear magnetic resonance (SSNMR)**

^14^N SSNMR spectra were measured at a Larmor frequency of 10 kHz with Bruker AVIII 400. The resonance frequency was referenced with respect to NH_4_Cl as a reference sample.

**Time-resolved photoluminescence (TRPL) spectroscopy**

Photoluminescence spectra and corresponding excitonic lifetimes were obtained from a bespoke micro-photoluminescence setup, in which a frequency-tripled Ti-Sapphire laser (λ = 266 nm, pulse duration = 120 fs, repetition rate = 76 MHz) was directed onto the sample. Time-resolved measurements were performed under a pulse-picking mode in order to tune the interval between two consecutive measured laser pulses (100 ns), which allowed the excited charge carriers to fully relax to the ground state (i.e., signal decaying to zero) before next pulse arrived. The spectrometer was used as a monochromator before passing the selected signal to an avalanche photodiode (APD) detector with an instrument resolution of ~50 ps connected to a time-correlated single-photon counting module.

The exciton lifetime is obtained by fitting corresponding background-corrected PL spectra with a bi-exponential decay function in the form

$y=A_{1}e^{-x/t_{1}}+A_{2}e^{-x/t_{2}}+y_{0}$.

**Electron paramagnetic resonance (EPR) spectroscopy**

Electron paramagnetic resonance (EPR) measurements were performed using a Bruker EMX EPR spectrometer (9.4 GHz) at the Centre for Advanced Electron Spin Resonance (CAESR), University of Oxford. All experiments were conducted at room temperature (293 K). X-band spectra were acquired over a magnetic field range of 1000 G. Signal intensity versus electron spin numbers were calculated from the double integration of a defined peak range of the spectra.

**Neutron powder diffraction (NPD)**

NPD was conducted at WISH beamline, ISIS Neutron and Muon Source, Rutherford Appleton Laboratory, UK. The WISH instrument is a time-of-flight cold neutron diffractometer, primarily designed for powder diffraction at long *d*-spacings (0.7–50 Å) for magnetic and large unit cell systems. WISH is equipped with pixelated ^3^He tube detectors covering almost the entire horizontal scattering plane (2*θ* range 10–170° on both sides). The scattering data are focused into five data sets at average 2*θ* values of 152.8°, 121.6°, 90.0°, 58.3°, and 27.1° of varying *d*-range and resolution.

Prior to the measurement, the perovskite sample was loaded into an 11 mm diameter vanadium can with quartz wool on top. The sample was degassed under high vacuum at 373 K to remove any guest molecules. The sample holder was then transferred to a vacuum chamber connected to a custom-made gas loading system. An NPD pattern of the de-solvated sample was first collected at 300 K for 20 min. After that, the He cryostat was used to cool the sample can to 7 K, where the thermal motion of the sample and adsorbed NH_3_ molecules can be significantly reduced. High-resolution NPD of this sample was carried out at 7 K for 1 h to get good statistics.

TOPAS-academic software, containing Rietveld refinement methods, was used to analyse the structural information of the diffraction patterns. For structural refinement, the initial HPNO-300 models were based on crystallographic data of HPrNb_2_O_7_. The diffraction patterns were refined by optimisation of the scale factor and lattice parameters. The background was described by a shifted Chebyshev function. A back-to-back exponential convoluted with pseuduo-voigt peak shape function was used to describe the peak asymmetry in the time-of-flight (tof) data. N_2_H_4_ was treated as a rigid body using Cartesian coordinates to reduce the number of variables. The refined structural parameters for all atoms of each pattern were the coordinates (x, y, z) and isotropic displacement factors (Beq). The quality of the Rietveld refinements of diffraction data was confirmed by low values of goodness-of-fit and R factors (R_wp_) and a well fitted pattern with acceptable Beq within experimental errors.

**High-angle annular dark-field scanning transmission electron microscopy (HAADF-STEM)**

TEM images were collected by using a Philips Analytical FEI Tecnai 30 electron microscope operated ultrasonically, dropped and dried on copper grid with lacy films. HAADF-STEM images were obtained on a double spherical aberration-corrected S/TEM Thermofisher Spectra 300 system at 300 kV with a field-emission gun. The probe convergence angle was 24.5 mrad, and the angular range of the HAADF detector was from 79.5 mrad to 200 mrad.

STEM–EDS mapping results were obtained by a Bruker Super-EDX 4 detector system.

**X-ray pair distribution function (XPDF)**

All the XPDF data was collected on BL08W at Spring-8, Japan.

The refinements of the XPDF were performed using the software PDFgui, where the scale factor, lattice parameters, dampening factor, broadening factor and atom occupancy were set to be refined. The goodness of the fit is given by:

$$R_{w}=\sqrt{\frac{\sum_{i=1}^{n} w(r_{i})\left[ G_{\mathrm{obs}}\left( r_{i} \right)-G_{\mathrm{calc}}\left( r_{i} \right) \right]^{2}}{\sum_{i=1}^{n} w(r_{i}){G_{\mathrm{obs}}}^{2}\left( r_{i} \right)}}$$

As a first attempt, HPNO perovskite model was refined by varying the cell parameter, an overall scale factor, metal and oxygen isotropic mean square displacement. Dealing with high temperature, 10 different models of HPNO were tried with oxygen vacancy value from 0.1% to 10%, until obtaining an optimal R_w_ value.

**Catalytic evaluation for NH_3_ decomposition**

The NH_3_ thermal decomposition was carried out in a purposely built continuous flow fixed-bed reactor with a computer-controlled auto-sampling system. Typically, 50 mg of catalyst was loaded in the centre of the quartz tube sandwiched with quartz wools. Prior to the reaction, the catalyst was treated under N_2_ gas flow for 30 min. The gas was switched to NH_3_ stream and then the catalyst bed was adjusted to the target reaction temperature. All the activity tests were conducted in the temperature range of 300 - 550 °C with varied WHSV under atmosphere pressure. After the stabilization, the gas composition was analysed by an online gas chromatography (Agilent 7890 A) equipped with TCD detector and a HayeSep Q column.

Photocatalytic NH_3_ was carried out in an autoclave under a solar simulator (1kW/m^-2^) as a light source. After placing the quartz loaded with photocatalysts in the autoclave, the autoclave was evacuated and purged 10 times with Ar gas. Then, the system was sealed with 6 bar of 240 cm^-3^ NH_3_ gas rather than a continuous process. The temperature was controlled by two heating bars assembled in the autoclave prior to the light illumination. The irradiation power in the centre of the autoclave window was calculated to be 80 mW. After light illumination for 1 h, the gas sample was analysed with a gas chromatograph (Agilent Technologies 7890B GC system) equipped with a thermal conductivity detector (TCD).

The reaction temperature in the photocatalytic activity test was continuously monitored and regulated using a thermal controller operating in proportional-integral-derivative (PID) mode. This setup maintains the bulk reaction temperature within ±0.1 °C of the setpoint by dynamically adjusting the heating input, in which the measured temperature reflects the bulk reactor environment, not the surface temperature of the catalyst particles.

Kinetic study was performed with varied temperatures to ensure that the reaction conditions was within the kinetic-controlled zone. The Arrhenius plots and activation energies for ammonia decomposition were calculated based on the activity evaluated under the same gas flow and compositions but at different temperatures. Arrhenius plots were obtained by linear fitting of *Ln* (reaction rate) versus 1/T. The apparent activation energy (*E*_a_) estimated from this plot, corresponding to the *E*_a_ of the rate-determining step in the overall chemical process.

**Statistics Analysis**

All the data normalisation, outlier evaluation was performed in Origin 2024. Each catalytic measurement has been repeated for three times in this work to give the error bar to represent standard deviation.

**
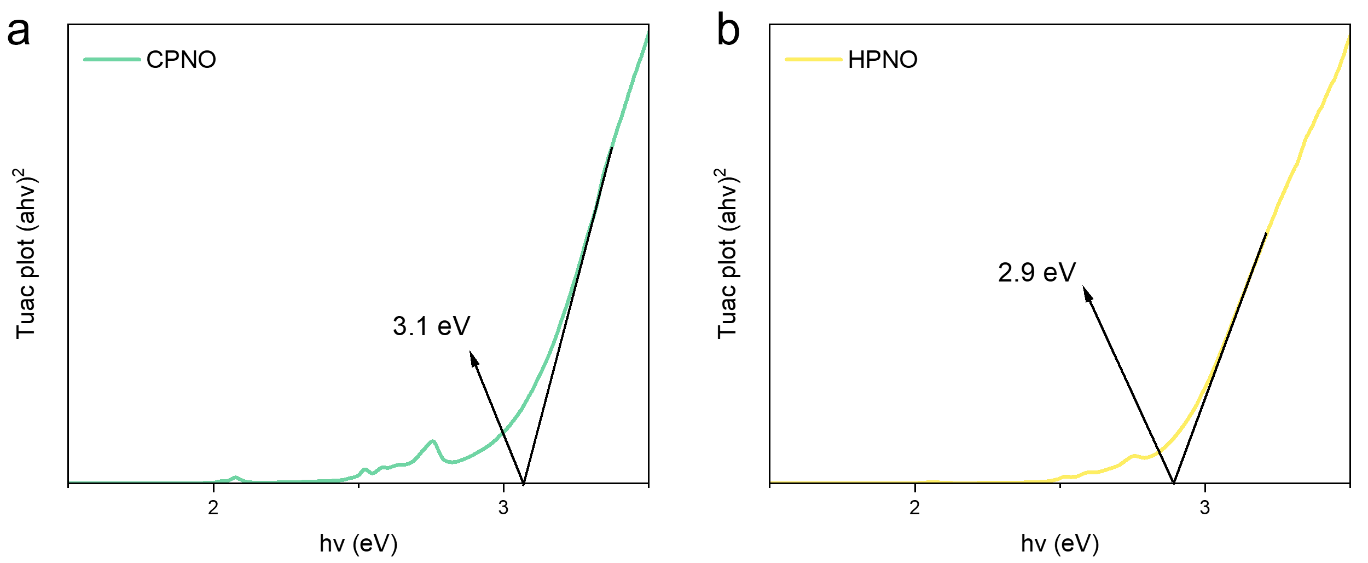
**

Figure S1: Tauc plot (ahv)^2^ of (a) CPNO and (b) HPNO.

**
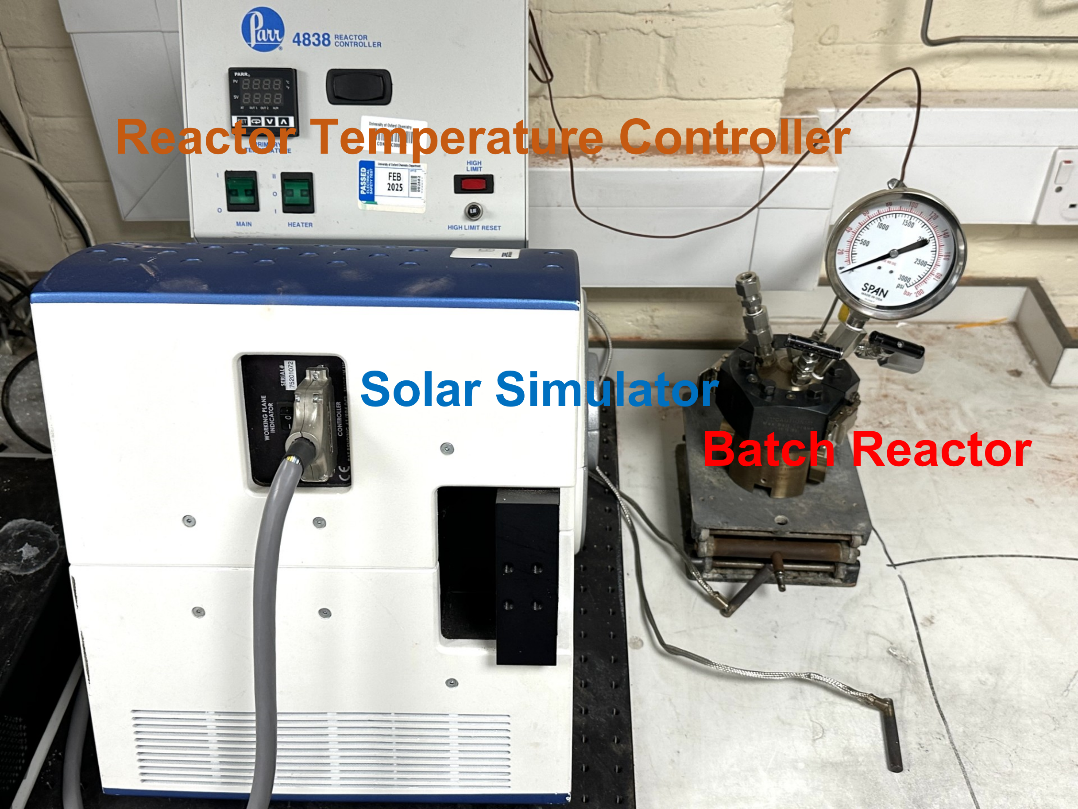
**

Figure S2: Experimental set-up figure. The batch reactor was heated by a thermal couple, where the temperature was precisely controlled by a reactor controller. In the experiments, the particulate photocatalyst powders are dispersed in a sealed batch reactor equipped with two opposing quartz windows of identical optical path length, allowing simulated solar light to pass and irradiate the photocatalyst powders. In addition, no detectable stray light exits the downstream window, indicating minimal irradiance loss.


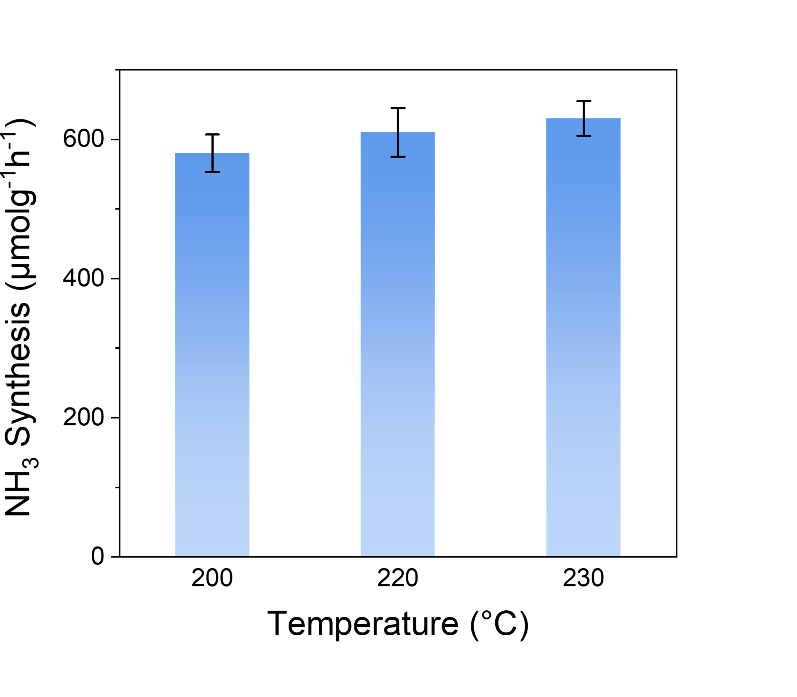


Figure S3: Evaluation of the reverse reaction (i.e., reaction between N_2_ and H_2_ for NH_3_ synthesis), in which the reactions were operated under 6 bar pressure of N_2_ and H_2_ gas mixture (9:1 v/v) in the batch reactor at different temperatures. (Error bars represent standard deviations)


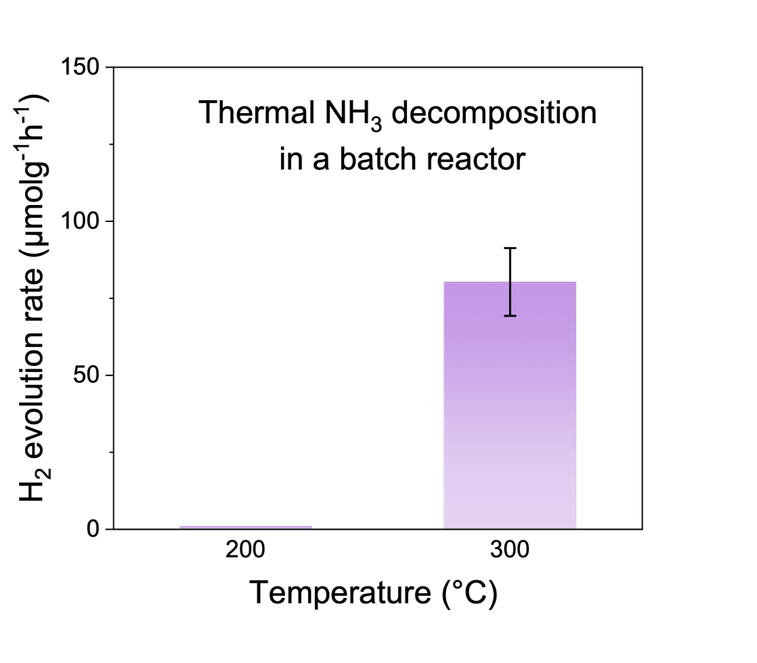


Figure S4: Thermal-only H_2_ evolution rate via NH_3_ decomposition at 200 and 300 ^o^C without light illumination using HPNO as the catalyst in the batch reactor. (Error bars represent standard deviations)


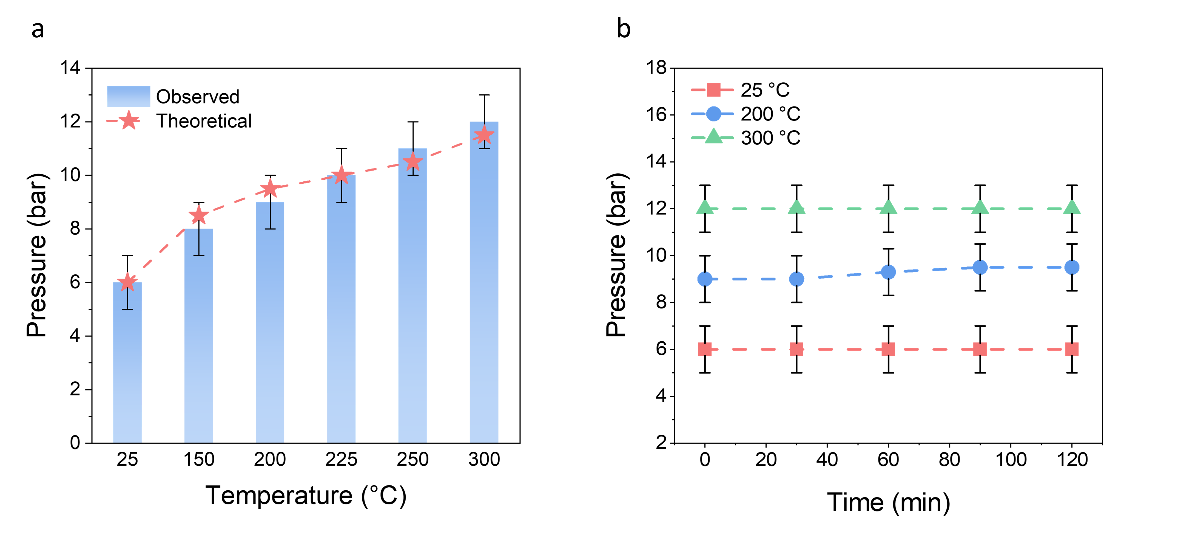


Figure S5: (a) Batch reactor pressure as the function of temperature. (b) Batch reactor pressure variation with different time under VeraSol solar simulator. (Error bars represent standard deviations)


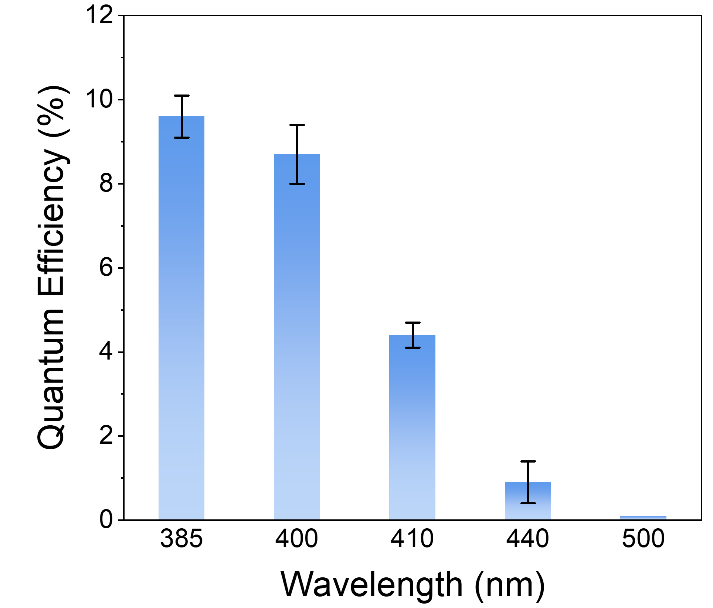


Figure S6: Quantum efficiency as a function of incident light wavelength during H_2_ evolution using HPNO as photocatalyst at 200 ℃. (error bars represent standard deviations)


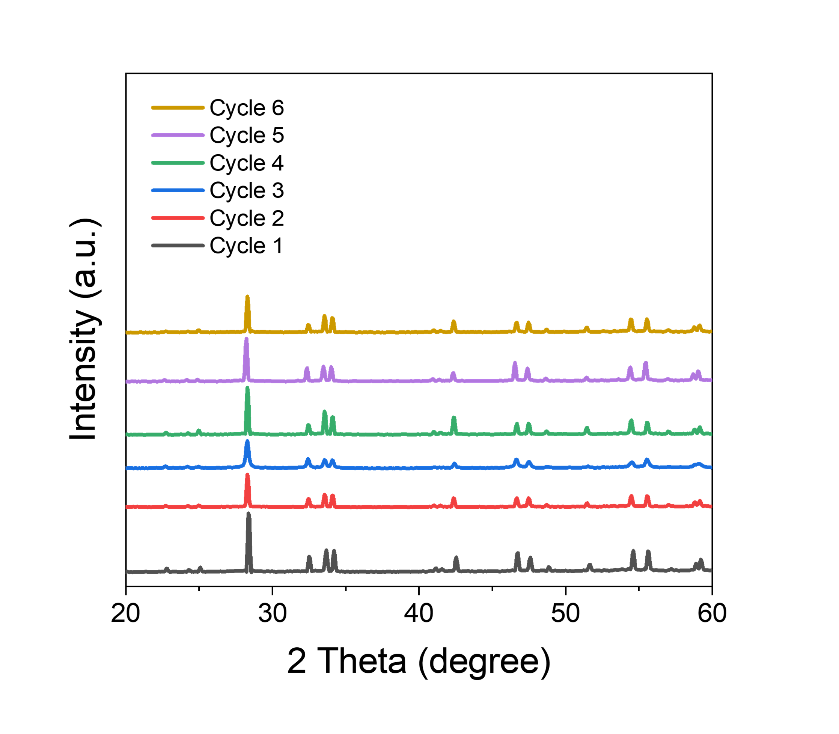


Figure S7: XRD pattern of HPNO photocatalyst after 6 times of photocatalytic ammonia decomposition tests.


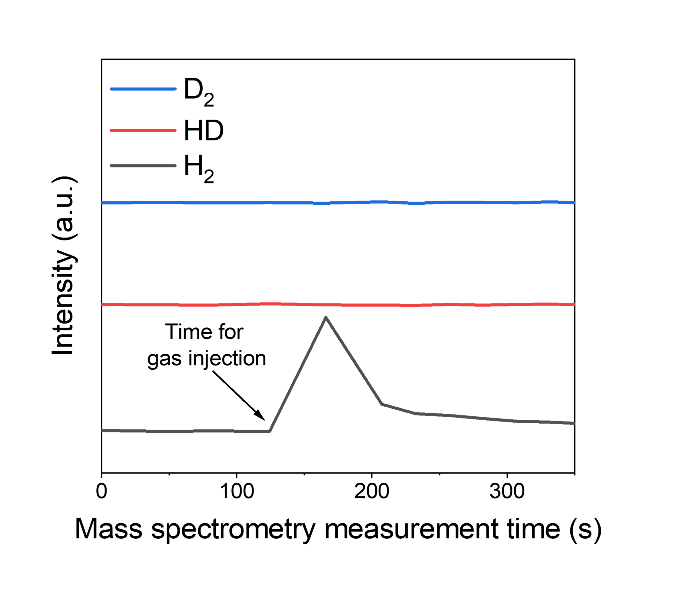


Figure S8: Time series signals of three gaseous molecules H_2_, HD, and D_2_ detected by a mass spectrometer, where DPrNb_2_O_7_ was used as the catalyst for NH_3_ decomposition.


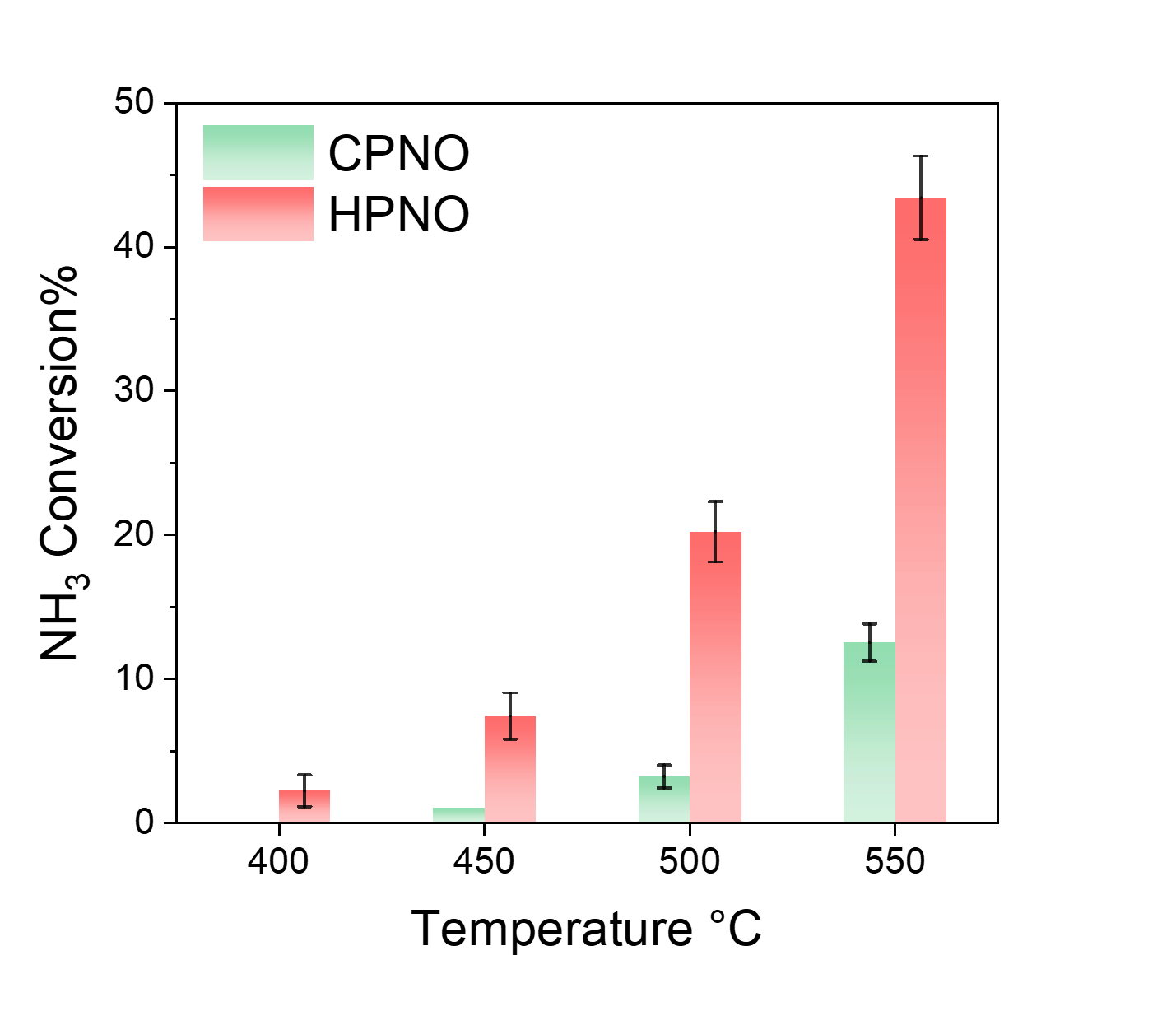


Figure S9: Ammonia conversion (thermal only) at different temperature over CPNO and HPNO, where weight hourly space velocity is maintained at **30,000 mL** g_cat_^-1^ h^-1^. (Error bars represent standard deviations)


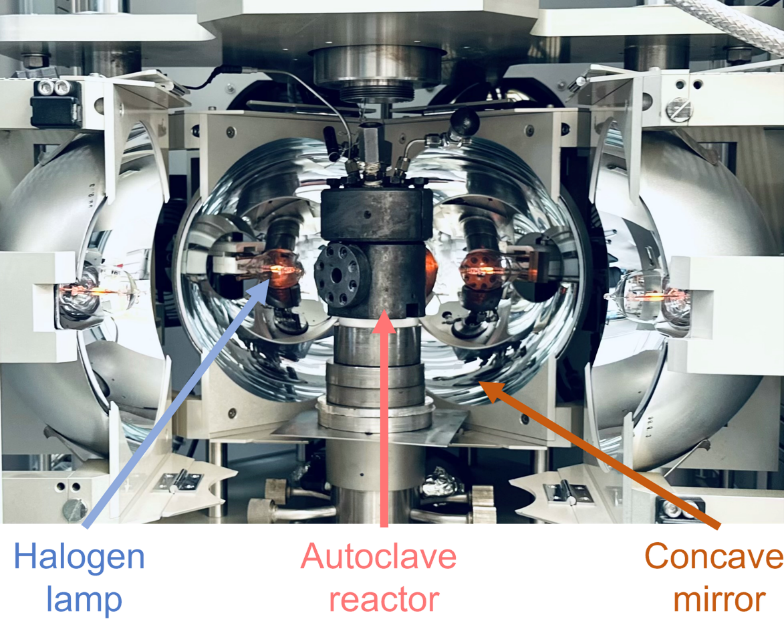


Figure S10: Photograph of a four-mirror floating-zone solar furnace from Crystal Systems used to mimic a solar concentrator to focus a light beam to provide both heat and photons without any other energy input from an electrical device.


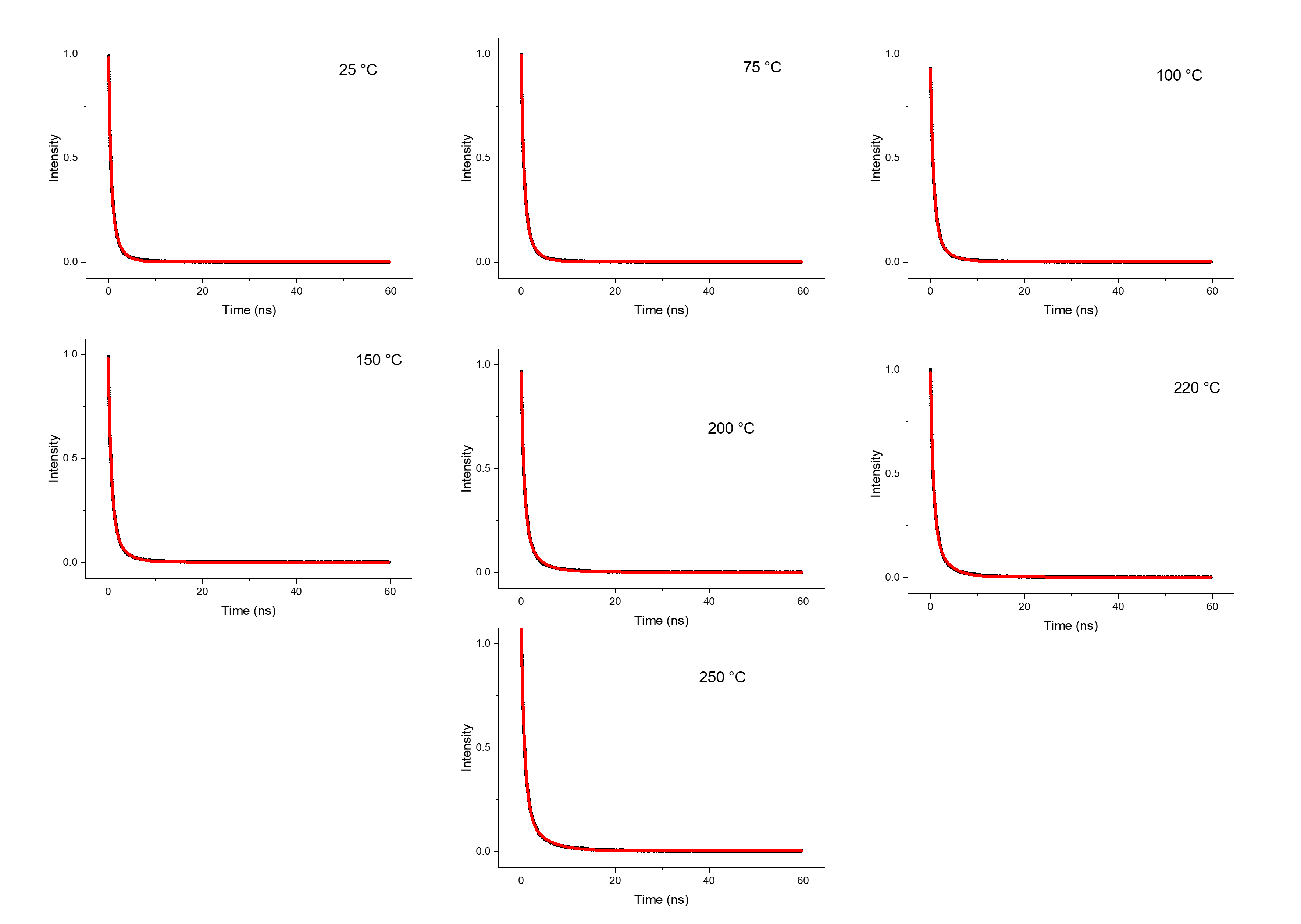


Figure S11**:** Exponential fittings of the TRPL spectra. The TRPL spectra were fitted using a biexponential decay function. The fitting parameters are listed in Table S1.


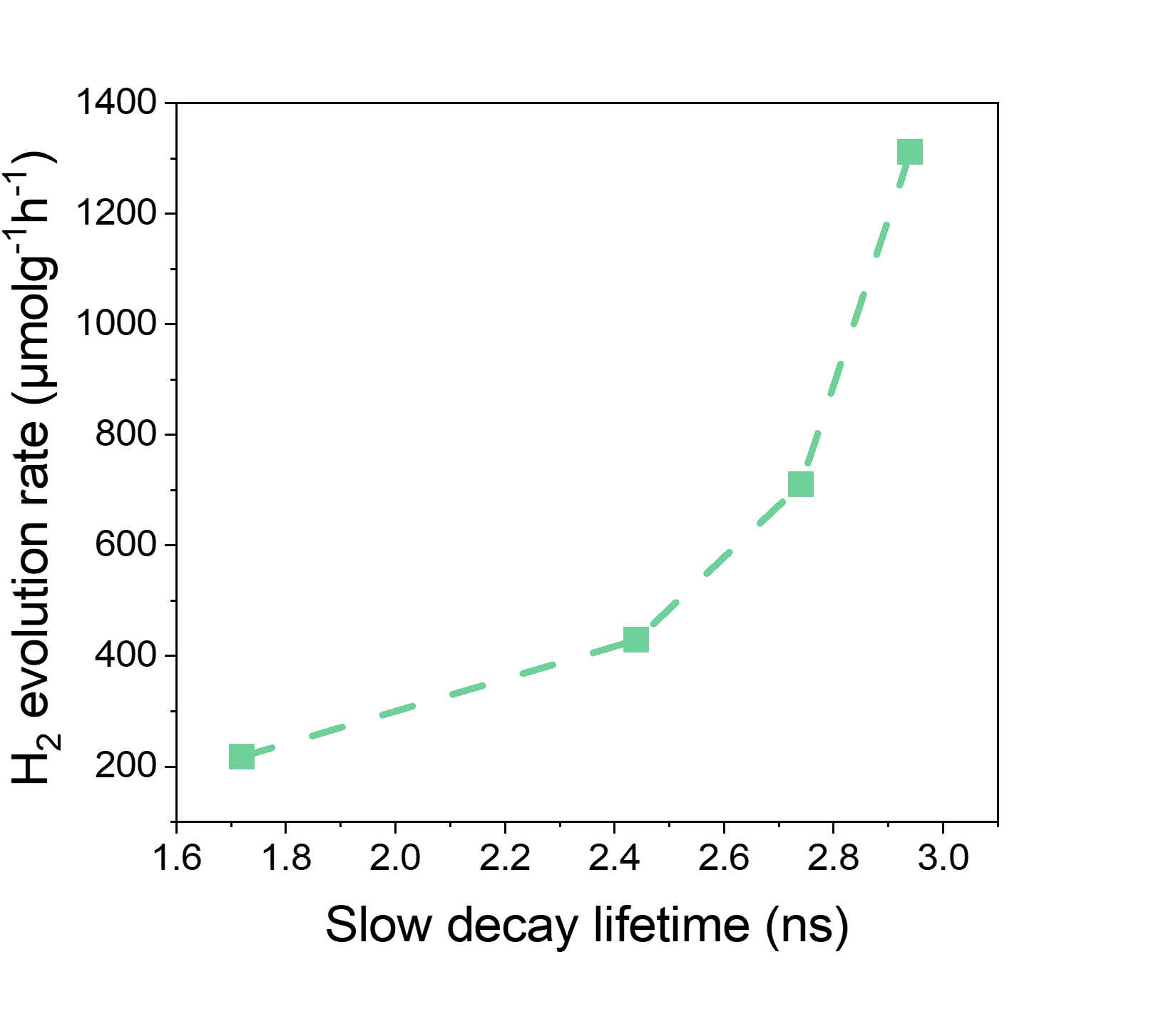


Figure S12. Photocatalytic activity versus charge carrier lifetime of HPNO.

**
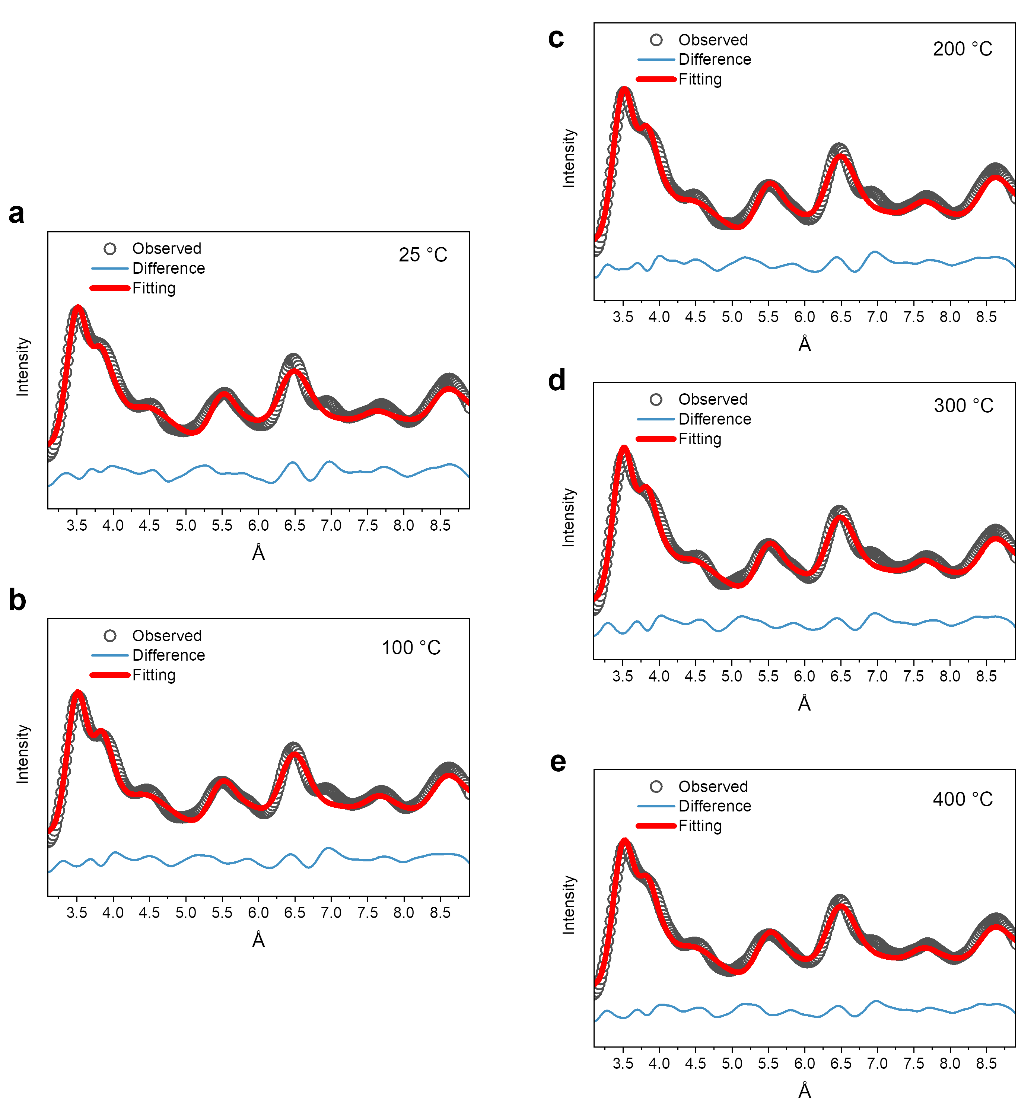
**

Figure S13: PDF refinement of HPNO under NH_3_ with increased temperature. Rw value: (a) 17.8%; (b) 17.9%; (c)18.7; (d)17.2%; (e) 16.1%.

**
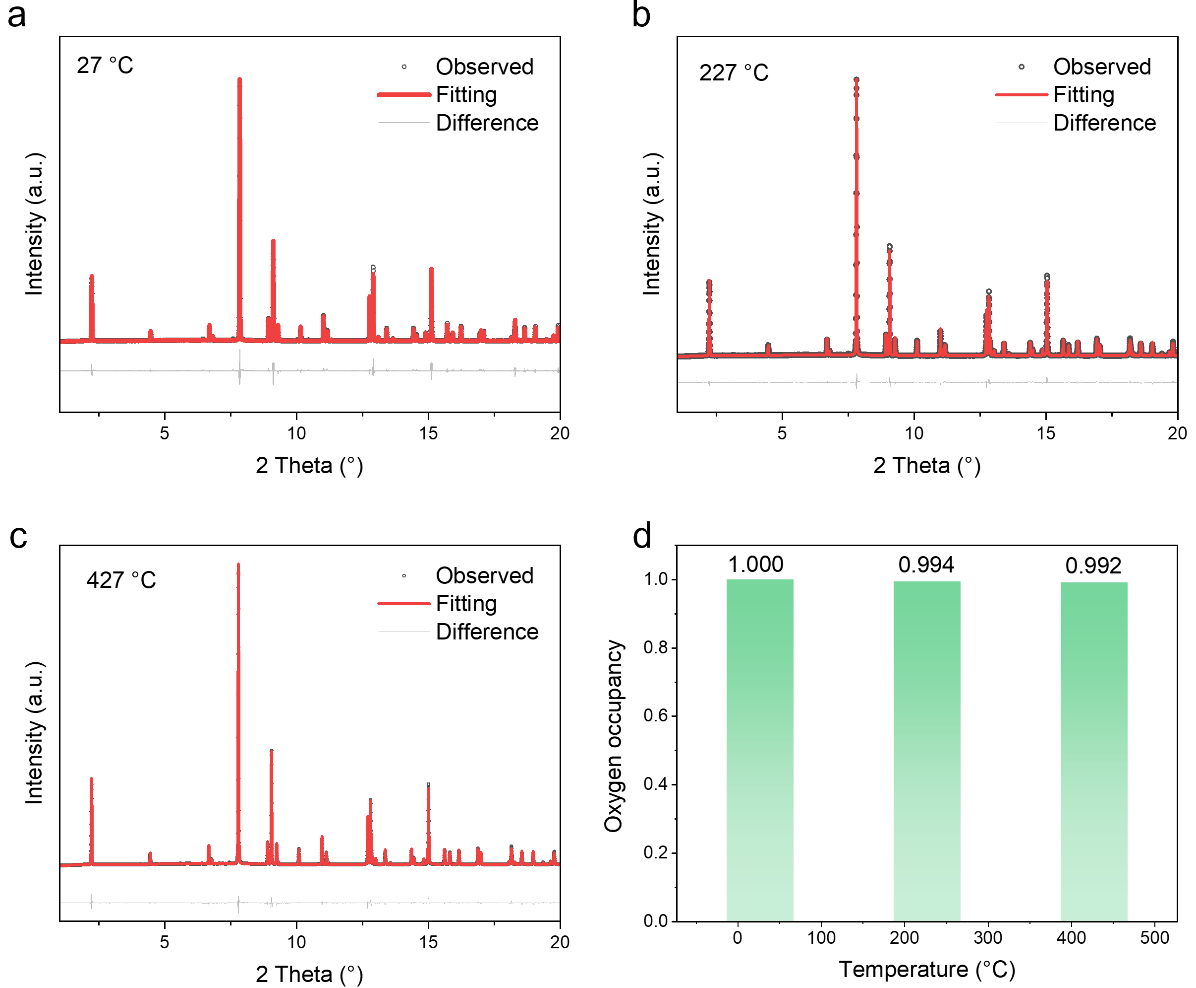
**

Figure S14: SXRD refinement of CPNO under air with increased temperature (a) 27 °C (Rwp = 11.0%), (b) 227 °C (Rwp = 7.2%), and (c) 427 °C (Rwp = 6.6%). (d) The oxygen occupancy value varies with different temperature.

**
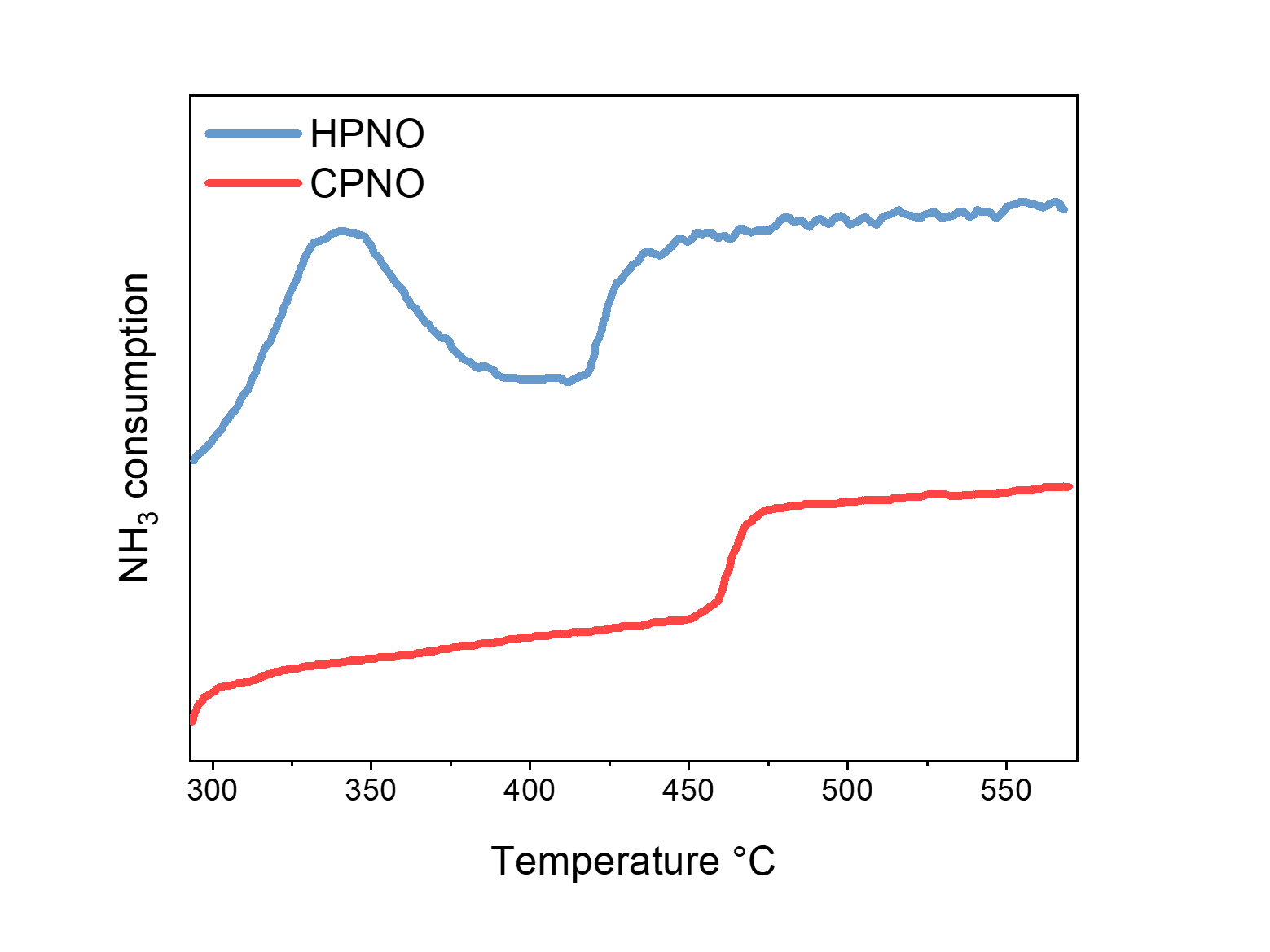
**

Figure S15**:** TPSR of HPNO and CPNO at different temperature with NH_3_ flow.


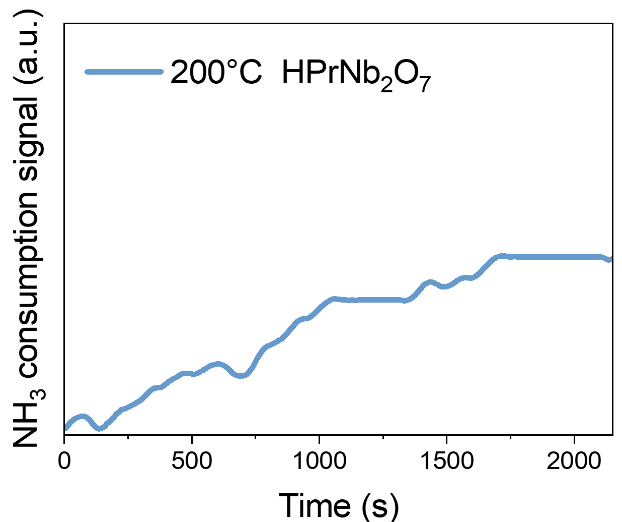


Figure S16: Ammonia consumption of HPNO at 200 °C at different time detected by TPSR.

**
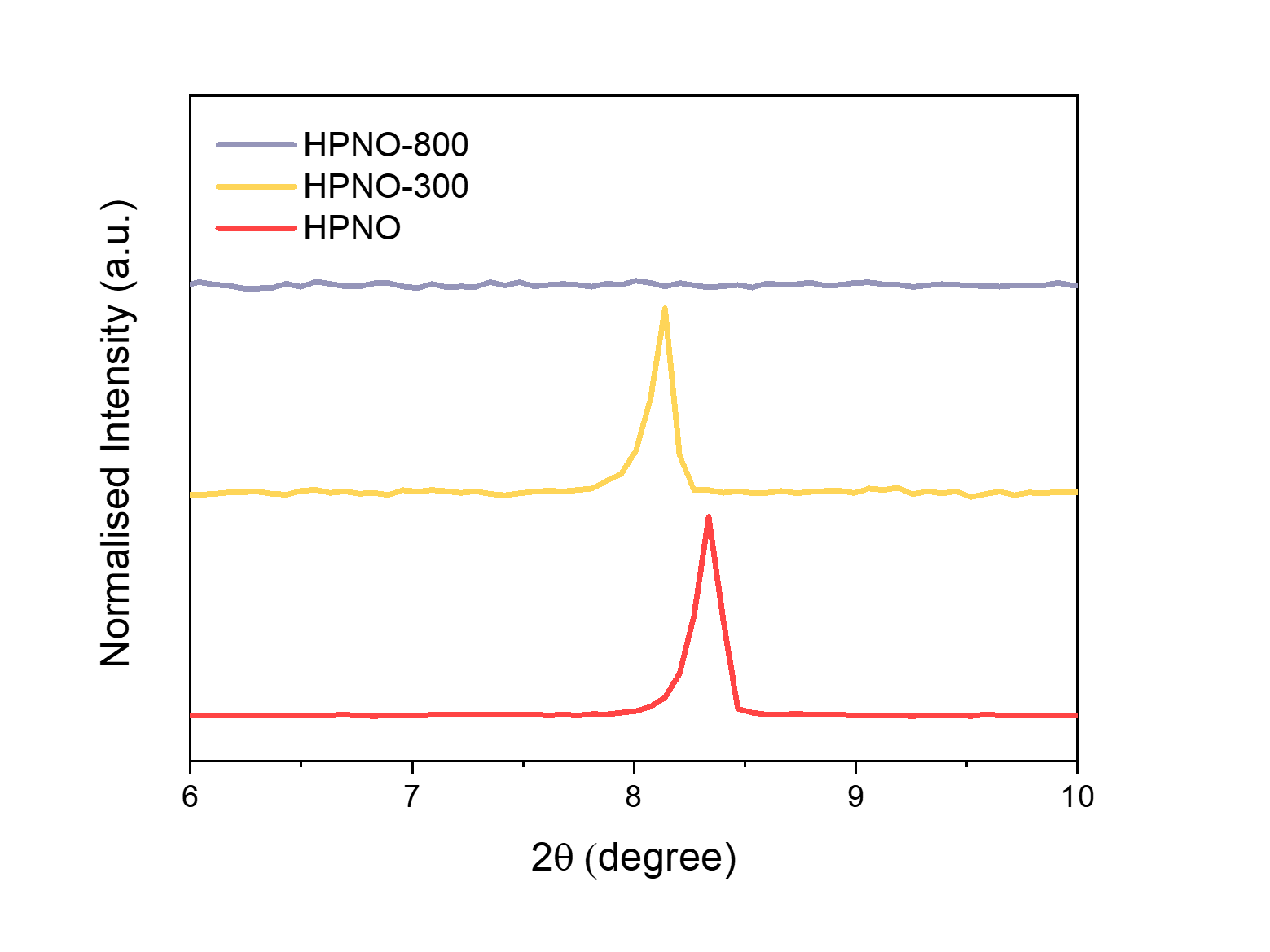
**

Figure S17: XRD pattern of HPNO, HPNO-300 and HPNO-800.


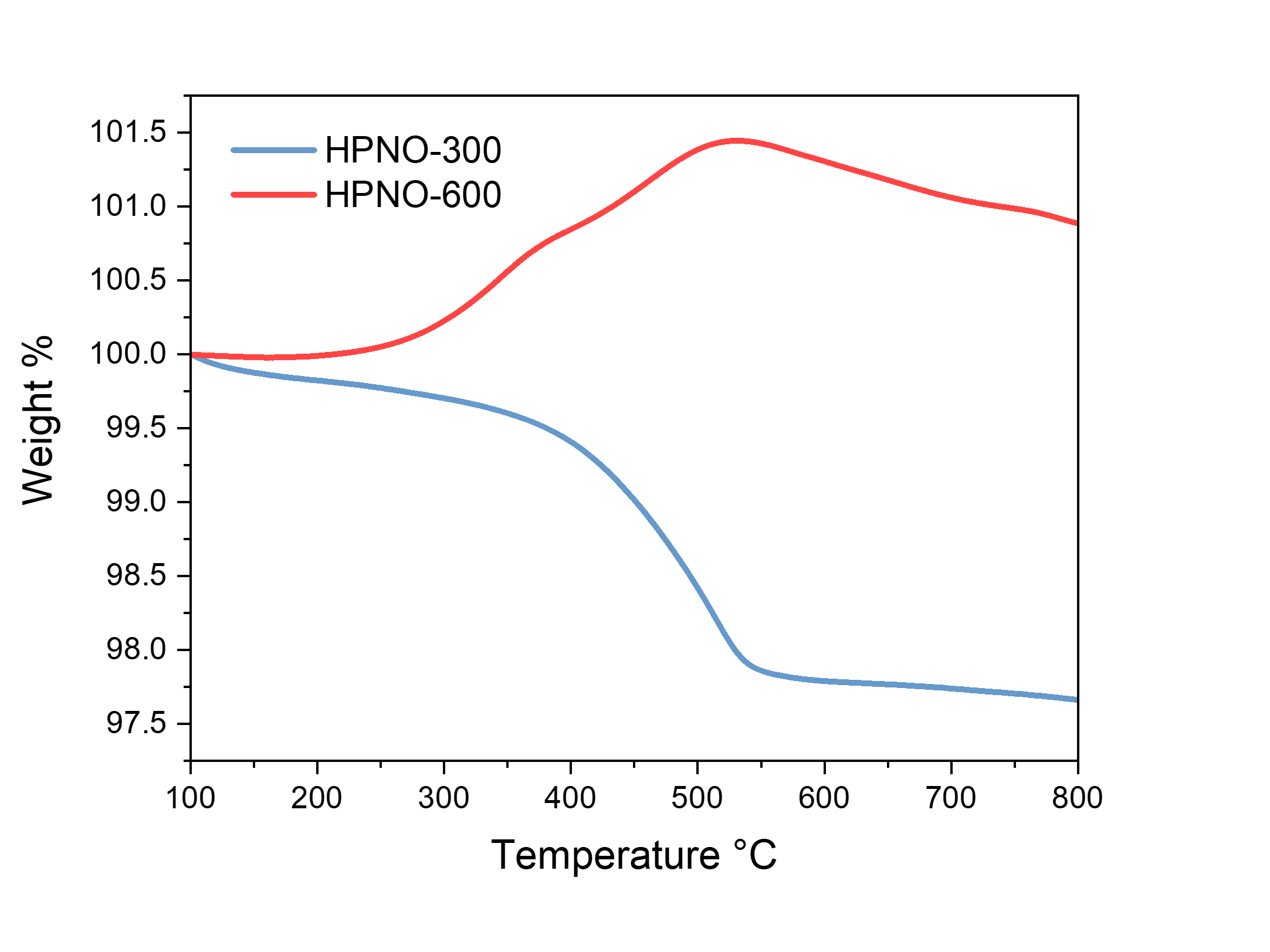


Figure S18**:** TGA curves under air flow for HPNO-300 and HPNO-600. Air flow rate: 100 mL min^-1^; ramp: 25 °C min^-1^.


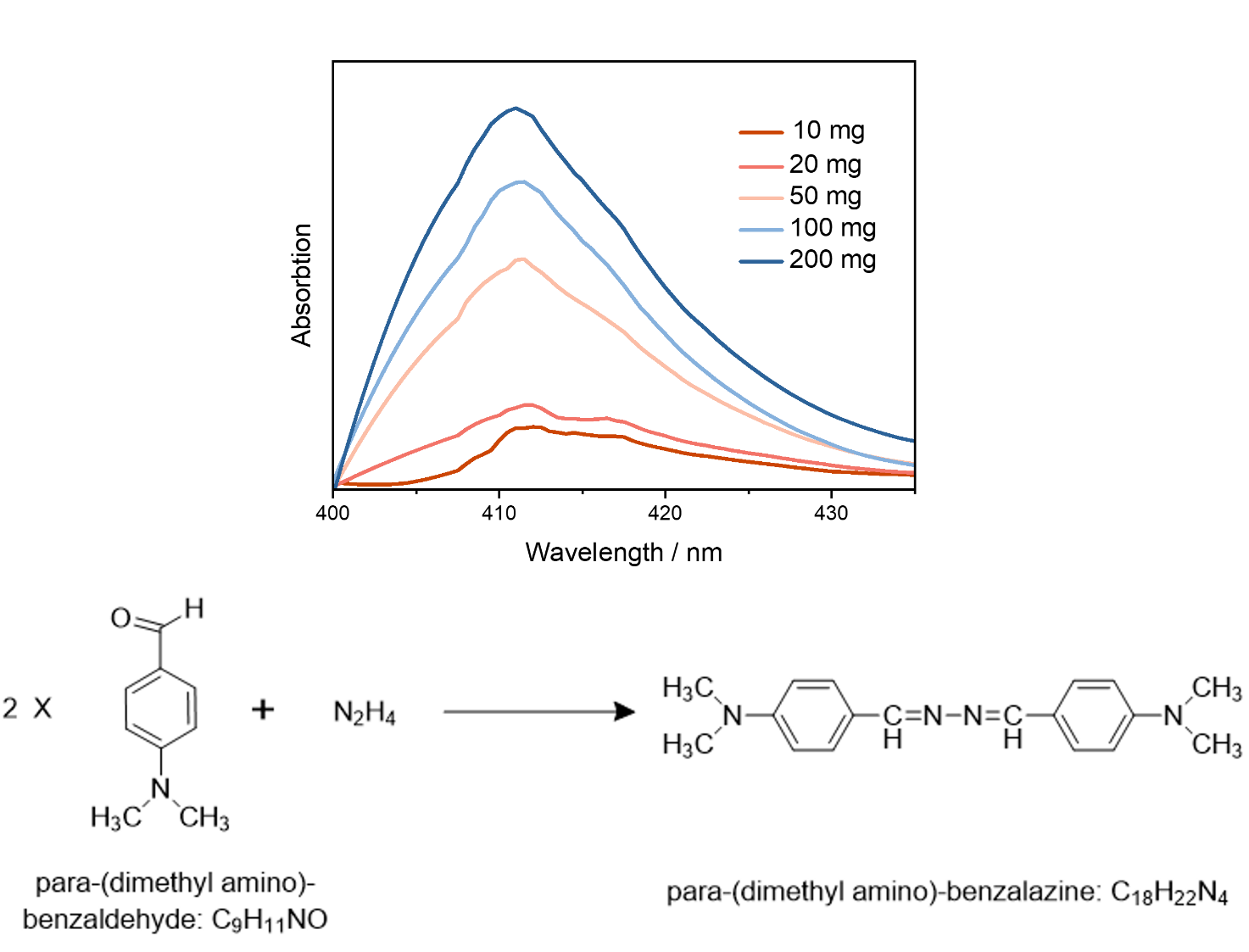


Figure S19: UV-vis absorption spectrum of *para*-(dimethyl amino)-benzaldehyde solution mixed with different amount of HPNO-300. Condensation reaction is between N_2_H_4_ and *para*-(dimethyl amino)-benzaldehyde. By the high-speed centrifuge, azine solution and HPNO-300 was well separated. Because no absorption was observed beyond 400 nm in UV-Vis spectrum of azine solution, which means HPNO-300 perovskite did not affect this absorption spectrum.

**
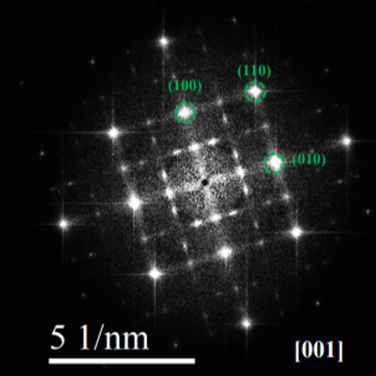
**

Figure S20: FFT image of HPNO-300 through [001] zone axis.


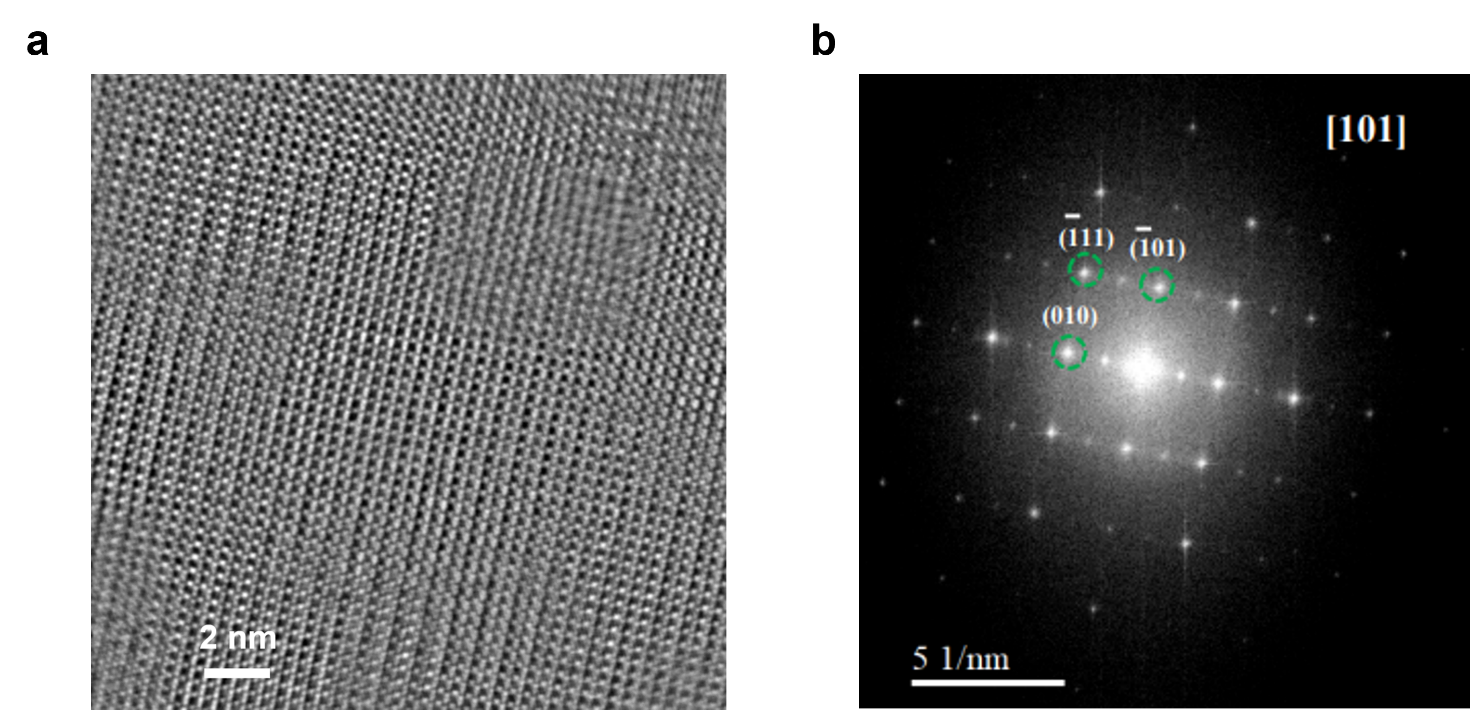


Figure S21: (a) HAADF-STEM images of HPNO-300 and its corresponding (b) FFT pattern through [101] zone axis.

**
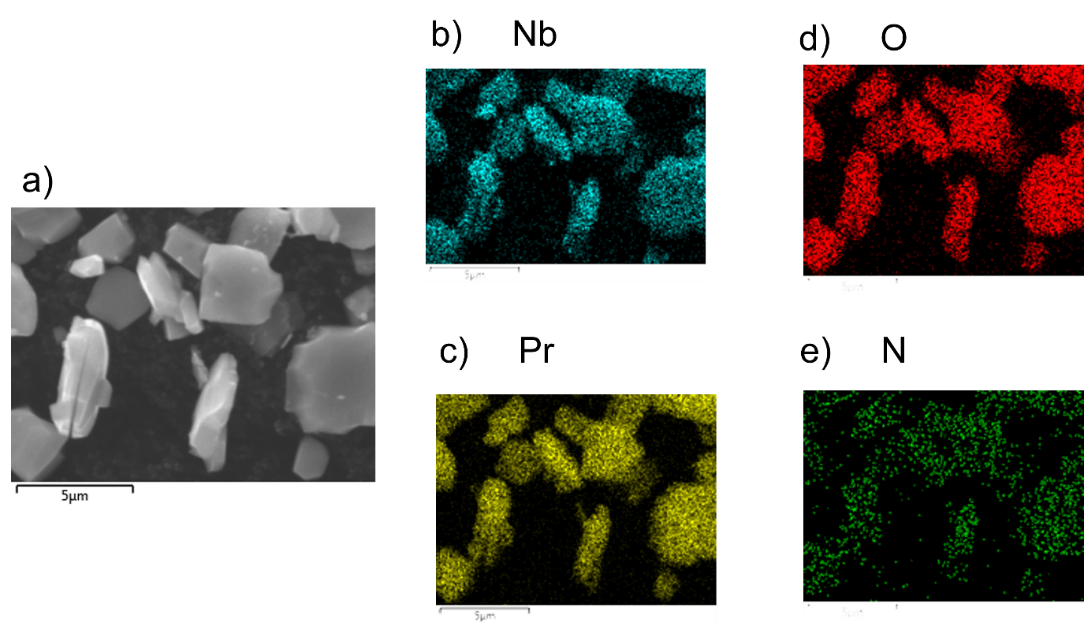
**

Figure S22: SEM images of HPNO-300 and the corresponding EDX mapping of Pr, Nb, O and N elements.


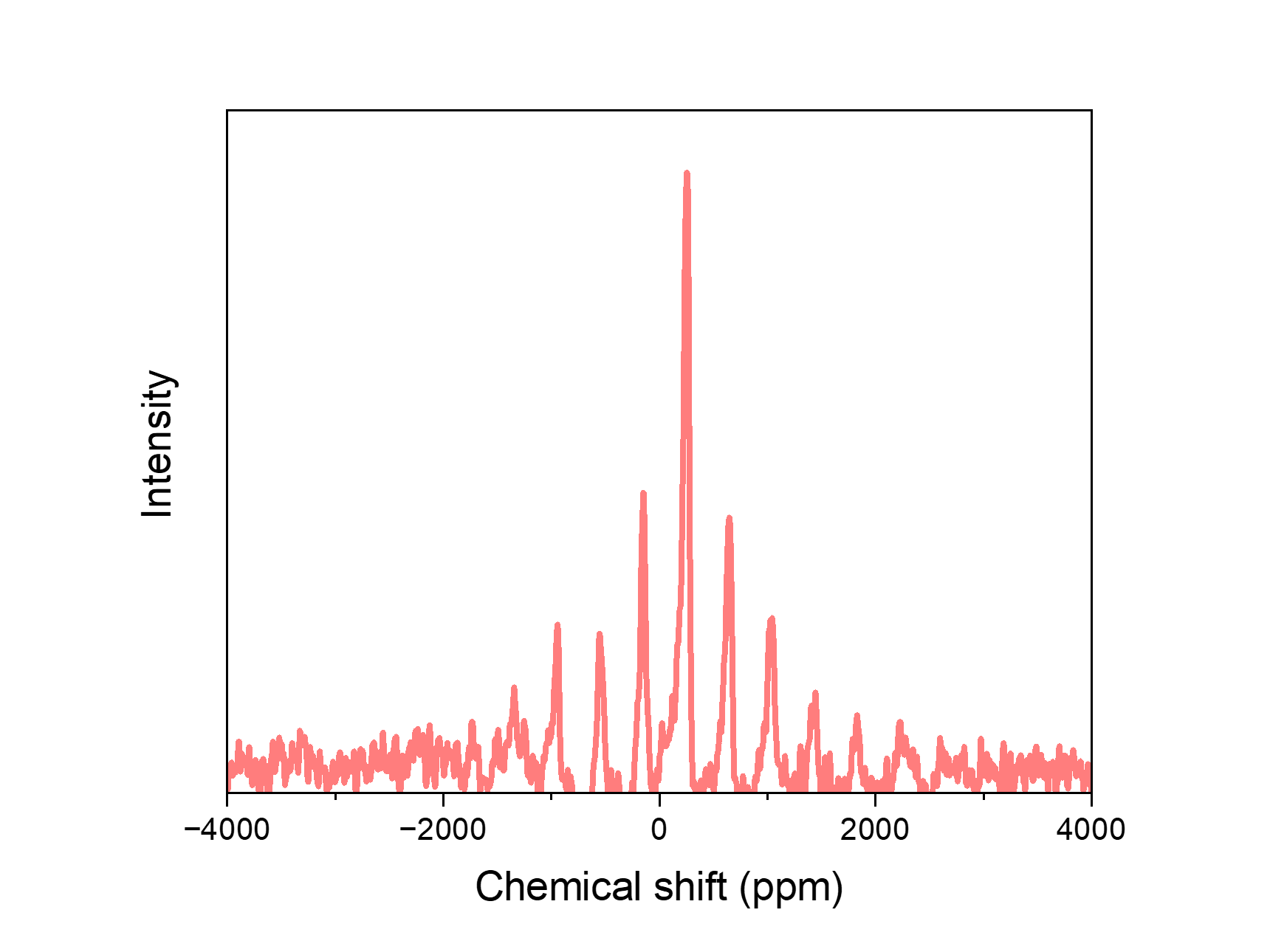


Figure S23: ^14^N ssNMR spectrum of HPNO-300. And the spectrum is dominated by a broad, asymmetric manifold spanning −2000 to 2000 ppm, characteristic of ^14^N nuclei (spin *I* = 1) experiencing strong quadrupolar interactions. These interactions arise from the electric field gradient at nitrogen sites, modulated by local symmetry distortions and dynamic processes within the perovskite lattice. The observed spectra breadth and multiplicity reflect the heterogeneous nitrogen environments, within f**irst-order quadrupolar splitting** dominates the line shape.


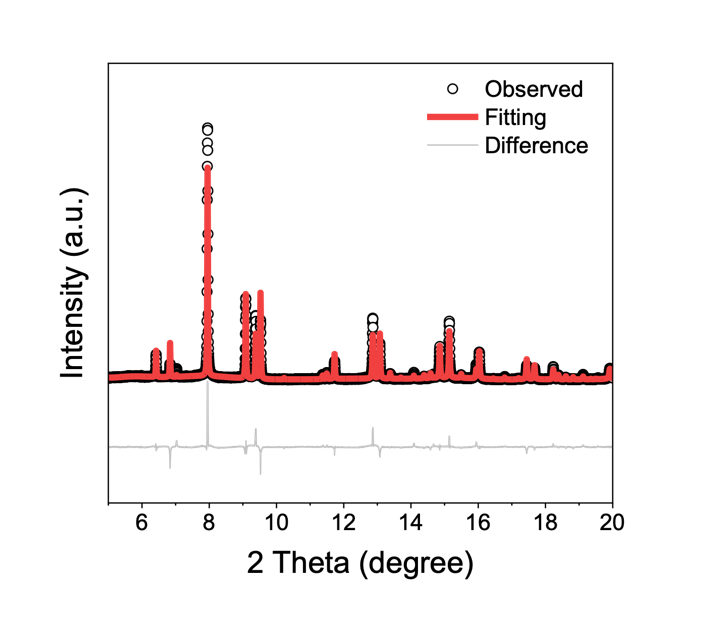


Figure S24: SXRD Rietveld refinement of HPNO-300 after illumination under 300 Xe lamp, where N_2_H_4_ could not be observed at the interlayer space. R_wp_ = 14.9%.


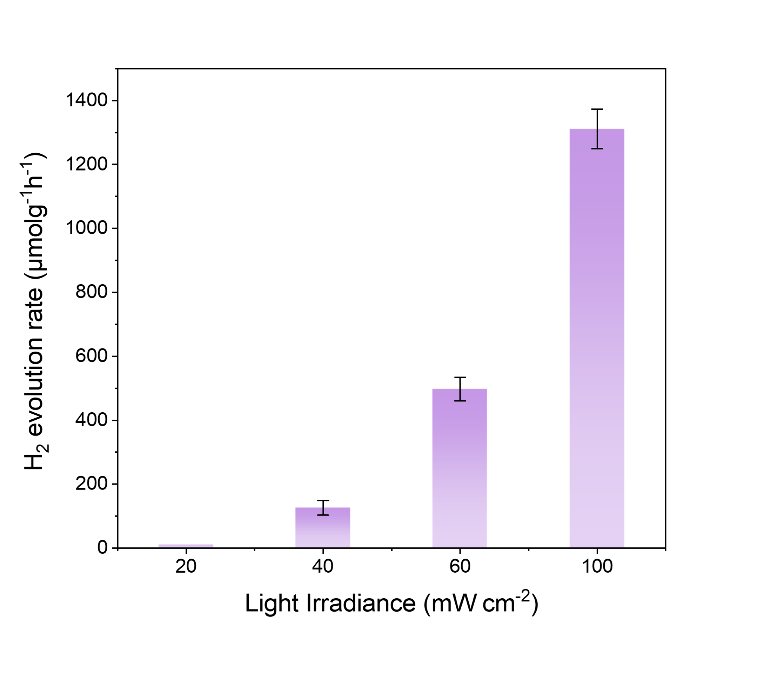


Figure S25: H_2_ evolution rate as a function of incident light intensity during NH_3_ decomposition using HPNO as photocatalyst at 200 ^o^C. (error bars represent standard deviations)

Table S1**:** Fitting parameter of TRPL spectra of HPNO at different temperature.

| Temperature/°C | t1 (ns) | A1 (%) | t2 (ns) | A2 (%) | t_ave_ (ns) |
| --- | --- | --- | --- | --- | --- |
| 25 | 0.46 | 66.3 | 1.72 | 33.7 | 0.88 |
| 75 | 0.59 | 74.3 | 2.09 | 25.7 | 0.98 |
| 100 | 0.65 | 75.6 | 2.44 | 24.4 | 1.09 |
| 150 | 0.70 | 75.8 | 2.74 | 24.2 | 1.19 |
| 200 | 0.66 | 75.1 | 2.94 | 24.9 | 1.23 |
| 220 | 0.68 | 75.5 | 3.12 | 24.5 | 1.26 |
| 250 | 0.79 | 80.2 | 4.19 | 19.8 | 1.46 |

Table S2: Thermally assisted photocatalytic NH_3_ decomposition activity comparison.

| **Sample** | **Light Source** | **Power(W)** | **Wavelength (nm)** | **Temperature (°C)** | **HER (μmolg^-1^h^-1^)** | **Ref** |
| --- | --- | --- | --- | --- | --- | --- |
| HPNO | Solar simulator | 0.08 W | 400-1000 | 25 | 218.7 | This work |
| HPNO | Solar simulator | 0.08 W | 400-1000 | 200 | 1311.2  (8.2*10^4^ μmol m^-2^ h^-1^) | This work |
| Ce 1.2%, TiO_2_ | UV lamp | 8 W | 254 | 25 | 106.8 | [1] |
| Pt 0.3%, TiO_2_ | Xe lamp | 500 W | > 400 | 25 | 200.1 | [2] |
| Pt/Fe TiO_2_ | Mercury arc | 100 W | > 300 | 25 | 3.4 | [3] |
| Pt/TiO_2_ (A) | Xe lamp | 300 W | 200-2500 | 50 | 216.5 | [4] |
| Ni-1.4-MCN | Xe lamp | 300 W | > 420 | 52 | 35.6 | [5] |
| Ru 0.5%, Fe_2_O_3_ | LED | N/A | 400-700 | 24 | 156.3 | [6] |

Note S1: Examples for calculation of QE

The quantum efficiency was measured in the same batch reactor following the same procedure as a typical photocatalytic measurement (see Methods for more details). However, the batch reactor was irradiated by a VeraSol solar simulator equipped with band-pass filters of 385, 400, 410, 440, and 500 nm, respectively, for QE measurements. The relevant number of incident photons was calculated from the irradiance measured using a light meter at each wavelength. Quantum efficiency can be then calculated using the equation as follows:

*QE* (%) = (*Number of evolved H_2_ molecules* × 2) / *Number of incident photons* × 100%

An example is given below:

The hydrogen amount analysed by GC is 0.09 µmol for the QE measurement at 385 nm, corresponding to 5.4×10^16^ hydrogen molecules;

During a period of 1 hour, the energy of the light irradiation: W = P×t. With the bandpass filter of 385 nm, the light power was measured to be P = 0.16 mW, therefore, the energy W = 0.00016 × 3600 = 0.58 J, which contains 1.12×10^18^ photons of 385 nm. Thus,

*QE* (%) = (5.4×10^16^×2)/ (1.12×10^18^) ×100% = 9.6 %

Each QE test was repeated three times, then the average value and standard deviation were calculated.

**References**

1. Reli, M., Ambrožová, N., Šihor, M., Matějová, L., Čapek, L., Obalová, L., Matěj, Z., Kotarba, A. and Kočí, K., 2015. Novel cerium doped titania catalysts for photocatalytic decomposition of ammonia. *Applied Catalysis B: Environmental*, *178*, pp.108-116.

2. Obata, K., Kishishita, K., Okemoto, A., Taniya, K., Ichihashi, Y. and Nishiyama, S., 2014. Photocatalytic decomposition of NH_3_ over TiO_2_ catalysts doped with Fe. *Applied Catalysis B: Environmental*, *160*, pp.200-203.

3. Fuku, K., Kamegawa, T., Mori, K., & Yamashita, H. (2012). Highly Dispersed Platinum Nanoparticles on TiO_2_ Prepared by Using the Microwave‐Assisted Deposition Method: An Efficient Photocatalyst for the Formation of H_2_ and N_2_ from Aqueous NH_3_. *Chemistry–An Asian Journal*, *7*(6), 1366-1371.

4. Yuzawa, H., Mori, T., Itoh, H. and Yoshida, H., 2012. Reaction mechanism of ammonia decomposition to nitrogen and hydrogen over metal loaded titanium oxide photocatalyst. *The Journal of Physical Chemistry C*, *116*(6), pp.4126-4136.

5. Lin, J., Wang, Y., Tian, W., Zhang, H., Sun, H. and Wang, S., 2023. Macroporous carbon-nitride-supported transition-metal single-atom catalysts for photocatalytic hydrogen production from ammonia splitting. *ACS Catalysis*, *13*(17), pp.11711-11722.

6. Dzíbelová, J., Hejazi, S.H., Šedajová, V., Panáček, D., Jakubec, P., Baďura, Z., Malina, O., Kašlík, J., Filip, J., Kment, Š. and Otyepka, M., 2023. Hematene: A sustainable 2D conductive platform for visible-light-driven photocatalytic ammonia decomposition. *Applied Materials Today*, *34*, p.101881.
